# Study on indoor thermal environment and energy consumption of traditional dwellings of ethnic minorities in Sichuan plateau

**DOI:** 10.1038/s41598-025-93002-8

**Published:** 2025-03-08

**Authors:** Yan Zhang, Biao Wang

**Affiliations:** 1 School of Architecture and Civil Engineering, Chengdu University, Chengdu, China; 2 Key Laboratory of Spatial Intelligent Planning Technology, Ministry of Natural Resources, Shanghai, China; 3 School of Architecture, Soochow University, No.199, RenAi Rd. Industrial Park District, Suzhou, 215123 China

**Keywords:** Sichuan plateau, Thermal division, Indoor thermal environment, Building energy simulation, Carbon emission, Civil engineering, Energy and society, Sustainability

## Abstract

In this paper, field tests, questionnaire surveys, and DesignBuilder were used to analyse the indoor thermal environment and energy consumption of traditional houses in a traditional ethnic minority village of Western Sichuan Plateau of China, The results showed that during the summer test period, the outdoor temperature range was 9.3–7.8 °C and the relative humidity range was 53.5–67.4%, while the indoor temperature range of the tested room was 13.3–2.3 °C, and the relative humidity range was 69.1–83.0%. The humidity is high, and the thermal environment does not meet the requirement of local standard. Therefore, corresponding energy-saving optimization measures are proposed. In the winter heating building model data, compared with the heat load before optimization, the energy saving reaches about 56.5%. In addition, the carbon emissions and economic suitability of different heating methods were evaluated. Electric heating, coal-fired heating and biomass heating have payback periods of 11 years, 24 years and 6 years respectively. With perspective focusing on the special regional and ethnic characteristics of the plateau, this research aims to promote energy conservation and sustainable development of local traditional buildings of ethnic minorities, and help improve the living environment of the Sichuan Plateau. In the future, a long-term monitoring mechanism can be established to continuously track residential buildings after the adoption of optimization measures to evaluate the actual effect of these measures.

## Introduction

Globally, building energy consumption has become an important issue^1^. With the rapid development of society, building energy consumption has accounted for more than 30% of global total energy consumption and about 27% of global energy emissions^2–4^. Finding a balance between indoor comfort and energy efficiency and efforts to reduce the negative impact on the environment has become an important challenge for the global construction industry. In the context of increasing energy and environmental pressures, the importance of energy-saving and environmentally friendly sustainable buildings is particularly urgent and important. Sustainable building practices play an important role in mitigating climate change and improving the comfort of occupants around the world. By adopting advanced energy-saving technologies and design concepts, sustainable buildings can significantly reduce energy consumption and carbon emissions, thereby mitigating the impact of climate change. Tahmasbi et al.^5^ explore some of the latest technologies and strategic approaches to building energy-efficient façade designs that enable facades to respond dynamically to environmental conditions and improve thermal performance to achieve net-zero energy buildings. The study also discusses the challenges and future opportunities for the application of these technologies in different climates and urban environments, providing valuable insights for sustainable building development. Mousavi et al.^6^ applied Design Builder software to optimize the roof design and operation of buildings, maximizing energy efficiency and thermal comfort in buildings, and promoting an innovative approach to energy-efficient design for sustainable buildings. Shen et al.^7^ combine advanced BIM technology with intelligent algorithms to optimize building design with Design Builder simulation technology, improve indoor thermal comfort and the quality of life of occupants, and create a healthier and more comfortable indoor environment. Wang et al.^8^ used Design Builder to simulate energy consumption and thermal comfort at different set points and determine which combination is optimal. Typical cities in different climate zones in China were selected using machine learning to determine the optimal set points for heating, cooling, and ventilation during the design phase to improve the energy efficiency and indoor thermal comfort of residential buildings in China. Park et al.^9^ used microencapsulated phase change materials (MPCM) to manufacture artificial marble and analyzed its indoor environmental control and energy-saving effects through field tests in apartment buildings in South Korea, and the results showed that the building material was effective in maintaining indoor temperature stability, reducing energy consumption and improving comfort. However, despite the remarkable progress made in sustainable building technology and design concepts, the research on traditional residential architecture in specific geographical and cultural contexts, especially the traditional residential houses of ethnic minorities in the Sichuan Plateau, is still insufficient.

Different regional climatic conditions lead to great differences in the development of sustainable architecture in different regions^10–15^, and the traditional dwellings of ethnic minorities in the Sichuan Plateau have strong regional and historical characteristics, which are valuable non-renewable and irreplaceable historical and cultural heritage in China, and these buildings embody the wisdom of harmonious coexistence between local people and nature, and have extremely high historical and cultural value. However, due to the constraints of climate, topography, and economic development, the local area faces multiple severe challenges such as a lack of conventional energy supply, weak energy infrastructure, and insufficient utilization of renewable energy, including (1) low household energy efficiency. In the Sichuan Plateau, due to the availability of local resources and traditional habits, traditional biomass (firewood, straw, animal manure)^16^ energy is the main source of energy for residents, and the combustion efficiency of these traditional biomass materials is low, resulting in low energy efficiency. The inefficient combustion process will produce a large amount of smoke and harmful gases, resulting in poor indoor air quality. (2) Weak energy infrastructure. Due to the constraints of plateau topography and economic development level, it is difficult to apply centralized energy supply technology, power grid, gas, liquefied gas, and natural gas supply have not been able to spread to all towns and villages, and conventional energy supply is scarce, resulting in the energy problem in the region is still severe. (3) Architectural design lacks scientific and standardized, traditional residential energy consumption is high. Due to the limited economic conditions and unique climate and environment, most of the residential design and construction on the Sichuan Plateau are completed by local craftsmen and villagers relying on the experience left by their ancestors, traditional customs and culture, and personal intuition. Craftsmen are to a certain extent unable to take full advantage of new technologies, materials, and methods, limiting the comfort and energy-saving efficiency of their homes. (4) Insufficient utilization of renewable energy. There are significant differences in the use of energy between rural and urban dwellings, and renewable energy should be fully utilized to reduce the total energy consumption of buildings. The Sichuan Plateau is a typical severe cold climate zone with abundant solar radiation resources, which provides good conditions for traditional houses without auxiliary heat sources in severe cold areas to base themselves on renewable energy, use solar energy to solve the problem of winter heating and improve living comfort and environmental protection while reducing the total energy consumption of buildings. In summary, these challenges not only affect the quality of life and cultural heritage of residents but are also closely linked to environmental protection and sustainable development. While protecting architectural heritage, there is an urgent need to improve indoor comfort. Therefore, it is of great significance to study the energy consumption mode and thermal comfort of traditional residential buildings of ethnic minorities in the Sichuan Plateau, and to propose targeted energy-saving measures. The objectives of the study should address the challenges of limited energy resources and weak infrastructure, such as optimizing building design and adjusting material selection to reduce energy waste, and exploring and using renewable energy sources, such as abundant local solar energy, as an alternative to traditional high-energy consumption methods; Research and promote energy-saving technologies and equipment suitable for the Sichuan Plateau, such as high-efficiency thermal insulation materials, to reduce the energy consumption level of residential buildings; In the case of weak infrastructure, optimize the layout and functional design of residential buildings to maximize the use of existing resources and improve the comfort and energy efficiency levels of residential buildings.

The energy consumption and thermal comfort of traditional houses of ethnic minorities are not only related to the quality of life and cultural heritage of ethnic minority residents but also closely related to environmental protection and sustainable development. It is of great significance to study the energy consumption patterns of traditional ethnic minority houses, reveal the characteristics and laws of energy use, put forward improvement measures for energy waste, and realize the effective use of energy, to alleviate energy shortage and promote sustainable development^17–20^. Li et al.^21^ pointed out that traditional Tibetan houses show a response to the unique local climate and discussed the generation mechanism of the indoor thermal environment of traditional houses in Lhasa. Rijal et al.^22^ conducted thermal measurements and thermal comfort surveys of traditional houses in the harsh winter climate of the Himalayan region of Nepal and showed that the thermal adaptability of buildings and their inhabitants is important for energy-efficient building design. Significant energy savings can be achieved through passive building design and lower indoor temperature settings for heating. Wang et al.^23^ took a house in Lhasa, Tibet as the research object, obtained the indoor air temperature distribution through field measurements, discussed the influence of outdoor meteorological factors on the indoor thermal environment, and pointed out that it is of great significance to make full use of geographical advantages to improve the indoor environment of passive buildings in the plateau area, which is of great significance for building energy conservation and thermal design of passive buildings. Li^24^ proposed that in the Lhasa plateau area, the near-zero energy heating of rural residential buildings can be realized by adopting efficient passive design methods. The results can be used as a guide for energy-saving design of rural residential buildings in plateau solar radiation areas in severe cold regions of China. Studying the energy consumption and thermal comfort of traditional dwellings of ethnic minorities can not only improve the quality of life of residents, protect and inherit ethnic minority culture, but also promote technological innovation, energy conservation, and emission reduction, promote the sustainable development of the construction industry, and contribute to the construction of a better and more livable living environment^25–29^.

Although scholars from all over the world have actively explored the energy conservation and emission reduction of traditional houses, there are few studies on the energy consumption of traditional houses of rural ethnic minorities compared with those of urban houses, and there are some limitations and shortcomings. The above study^21^ mentions the use of quantitative methods for analysis but lacks an in-depth understanding of the actual situation of rural minority housing. Some studies^23^ only consider the influence of some factors on the indoor thermal environment but do not comprehensively and accurately consider other factors, and the research on the thermal insulation performance of traditional residential envelope structures and the thermal properties of building materials still needs to be deepened. Some studies^22^ focus on the problems and deficiencies in the design of residential buildings, such as building orientation, materials, roof design, etc. However, no specific measures were provided on how to improve these design issues, etc. In addition, there is a relative lack of interdisciplinary cooperation and exchange, which leads to the one-sidedness and limitations of the research results. There is a lack of research on the traditional dwellings of ethnic minorities in the Sichuan Plateau. Due to the complexity of the natural environment, regional cultural differences, and local cultural traditions, there are relatively few empirical studies on building energy consumption, and the construction level of human settlements lags significantly behind that of other regions. Therefore, this study aims to fill this knowledge gap, gain an in-depth understanding of the actual situation of ethnic minority residential buildings in the Sichuan Plateau through field investigation and analysis, and propose energy-saving measures to adapt to the local area, so as to provide guidance for the sustainable development of local ethnic minority residential buildings.

In this study, we obtained first-hand data on the indoor thermal environment of the traditional houses of ethnic minorities in the Sichuan Plateau, including indoor temperature, humidity, airflow velocity, and the temperature of each wall of the envelope structure, through field measurements and questionnaire surveys. These data provide a solid basis for subsequent simulation analysis. Through the energy consumption simulation software, the indoor thermal environment and energy consumption of residential buildings are accurately simulated by comprehensively considering the factors of building, environment, energy, and other factors, and the impact of different optimization and renovation measures on the indoor thermal environment and energy consumption is analysed, and the economic cost and feasibility analysis are carried out, so as to select the optimal solution. This will not only help improve the living comfort and energy efficiency of residential buildings but also effectively reduce the energy consumption costs of residential buildings and promote the sustainable development of the Sichuan Plateau.

The Chinese government has been committed to promoting the development of ethnic minority areas, including reform and innovation in the energy sector. Research on improving energy efficiency in the Sichuan Plateau can be strongly supported by policies and regulations. The problems in energy use in the Sichuan Plateau region are universal, such as weak energy infrastructure and low energy efficiency. Therefore, the technical achievements and solutions obtained in the research carried out in this area can also have certain applicability in other similar areas. As a unique geographical unit in China, the Sichuan Plateau can exchange and share its research and practical experience in energy efficiency improvement with similar regions in the world.

## Methodology and material

**Fig. 1:**
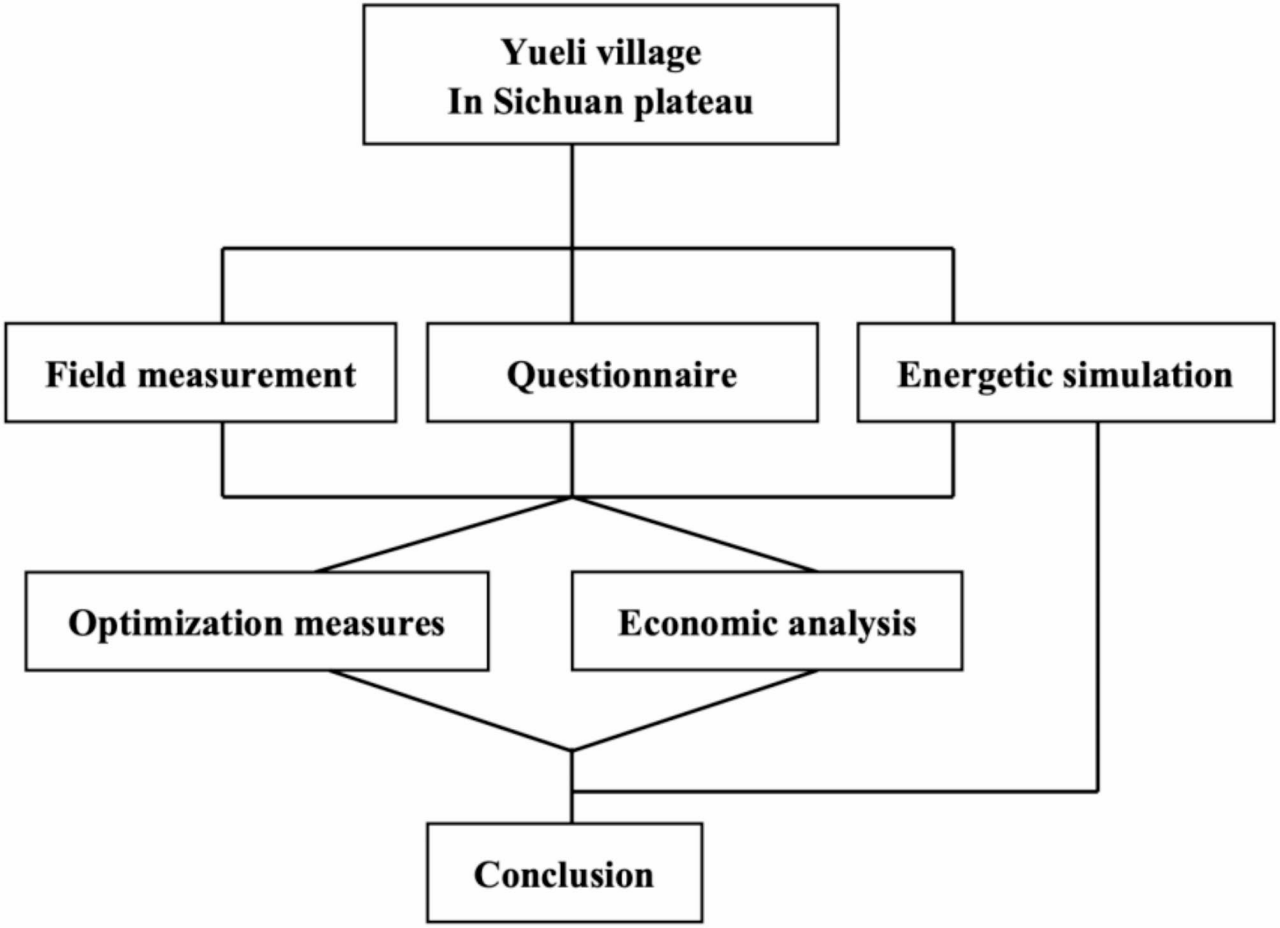
Research roadmap.

As shown in Fig. 1, the research method mainly uses field measurements, questionnaire surveys, and energy simulations to conduct an in-depth analysis of the factors affecting the energy consumption of residential buildings in Sichuan Plateau, and to measure and quantitatively analyse the indoor thermal environment of local traditional residential buildings. The key factors of high energy consumption are identified, and some energy-saving improvement and optimization measures and economic suitability analysis are proposed.

### Research object

**Fig. 2:**
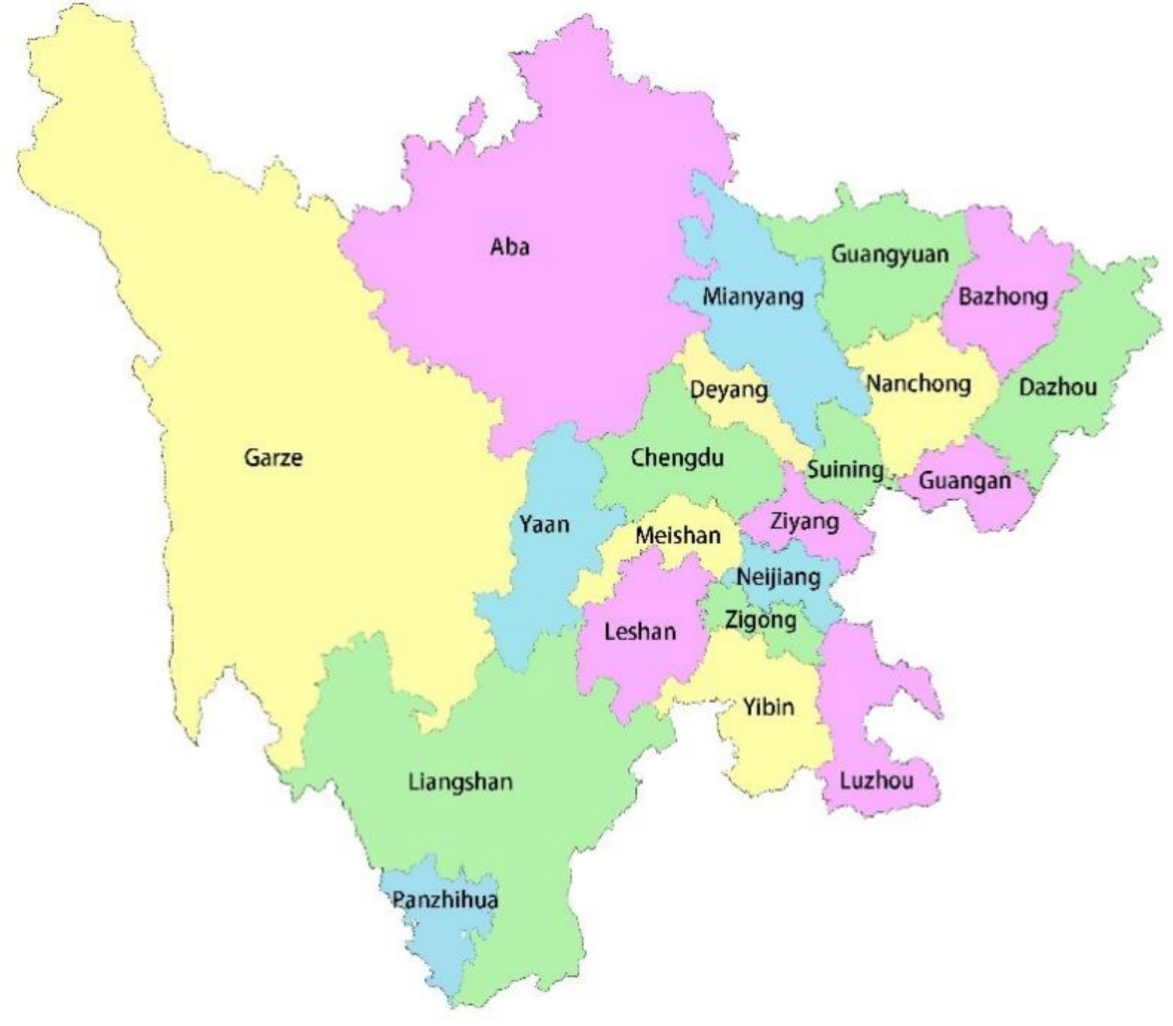
Map of Sichuan Province.

**Fig. 3:**
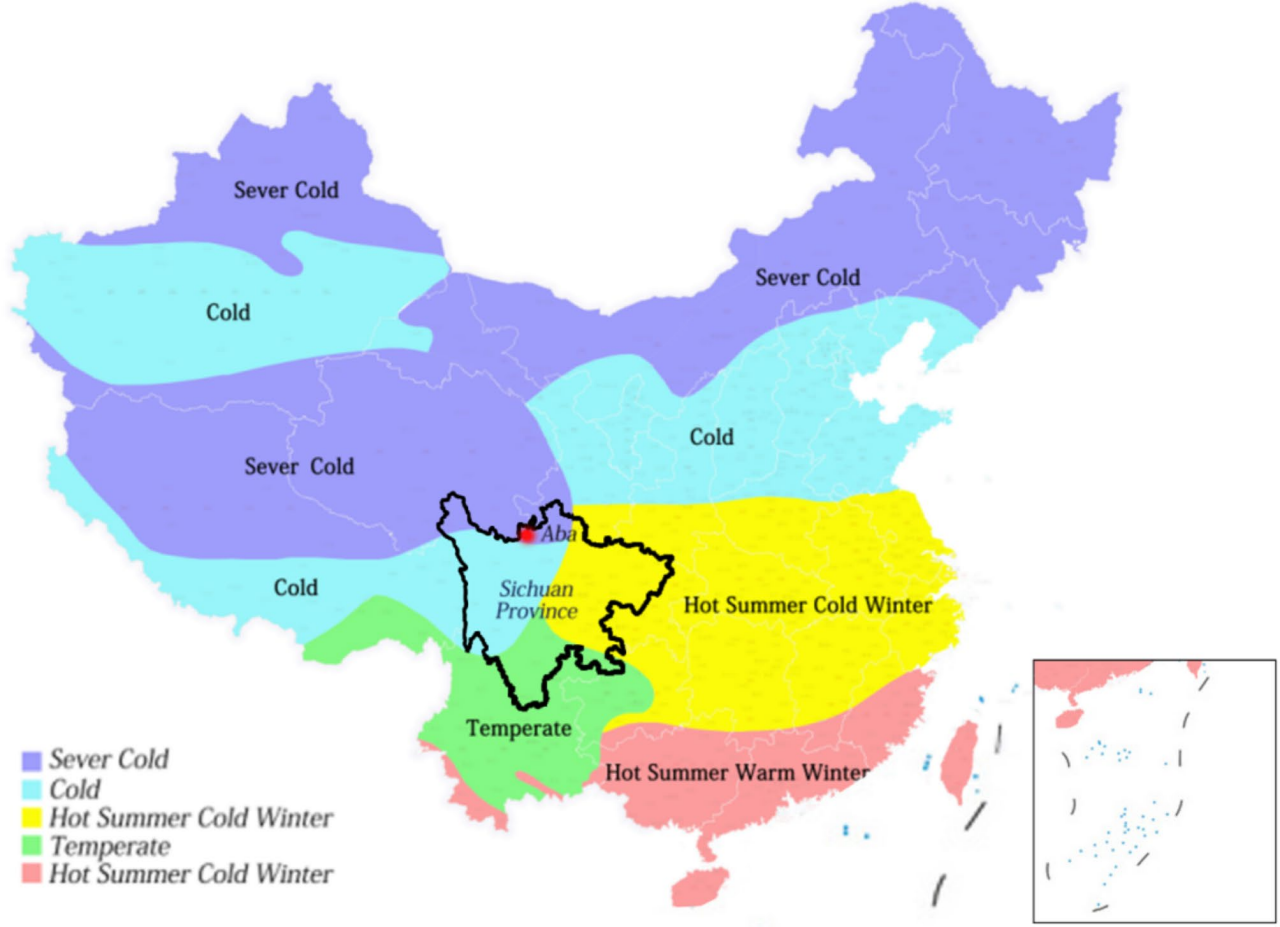
The location of Aba Prefecture and the climate division of China.

The Sichuan Plateau region is composed of three prefectures: Garze Prefecture, Aba Prefecture, and Liangshan Prefecture, as shown in Fig. 2, Aba is located in the Sichuan Plateau region, about 104.27° east longitude, 30.35° latitude, the highest altitude in the territory is about 6250 m, and the average altitude is 3500–4000 m. According to the Code for Thermal Design of Civil Buildings, there are five climatic zones in China, namely severe cold areas, cold regions, hot summer and cold winter regions, mild regions, and hot summer and warm winter regions^30^. Aba Prefecture is a severe cold area, as shown in Fig. 3. In this study, the traditional houses of Yueli Village, Wenchuan County, Aba Prefecture, were selected as the research object rather than the famous traditional settlements, which can better reflect the universality and authenticity of the research.

### Questionnaire

A random sample survey of long-term residents of Yueli Village, Wenchuan County, Aba Prefecture was carried out from July 19 to July 25, 2024 to evaluate the indoor environment of traditional houses. 135 questionnaires were issued, and 123 valid questionnaires were returned. The data are representative and can objectively reflect residents’ feelings about the indoor thermal environment. The interview content involves residents’ thermal comfort in the indoor environment, including the number of permanent residents, gender, age, living habits, construction area, building materials, etc. During the interview, respondents were asked about indoor temperature, humidity, air quality, and whether they felt cold, hot, stuffy, wet, and other symptoms. Respondents were also asked whether they used fans, air conditioners, or other equipment to regulate indoor temperature and humidity. In addition, respondents were also asked about their overall satisfaction and comfort with the indoor environment. The main contents of the questionnaire, most of which are open-end questions, are shown in Table 1. These questions can help us better understand the feelings and needs of residents for thermal comfort in the indoor environment.

**Table 1:** Main content of the questionnaire on residents’ thermal comfort.

Number	Questions
1	How old are you? Gender? Number of family members
2	What kind of energy is mainly used by households? Electricity, household coal, traditional biomass (firewood, straw, animal manure)
3	What is the wall material of the house? What are the layers and materials of window glass?
4	How is your current feeling?
5	How comfortable do you think of the current indoor temperature?
6	How do you want the current indoor temperature to change?
7	What do you think of the current indoor humidity?
8	How comfortable do you think of the current indoor humidity?
9	How do you want the current indoor humidity to change?
10	What do you think of the current indoor airflow situation?
11	How do you want to change based on your sense of wind?
12	Do you think the current indoor thermal environment is acceptable?
13	Where do you think the discomfort of the indoor environment comes from?
14	In summer, do you think the room needs to be dehumidified?
15	Do you have any suggestions to improve the comfort level of residential buildings?

### Field measurements

**Fig. 4:**
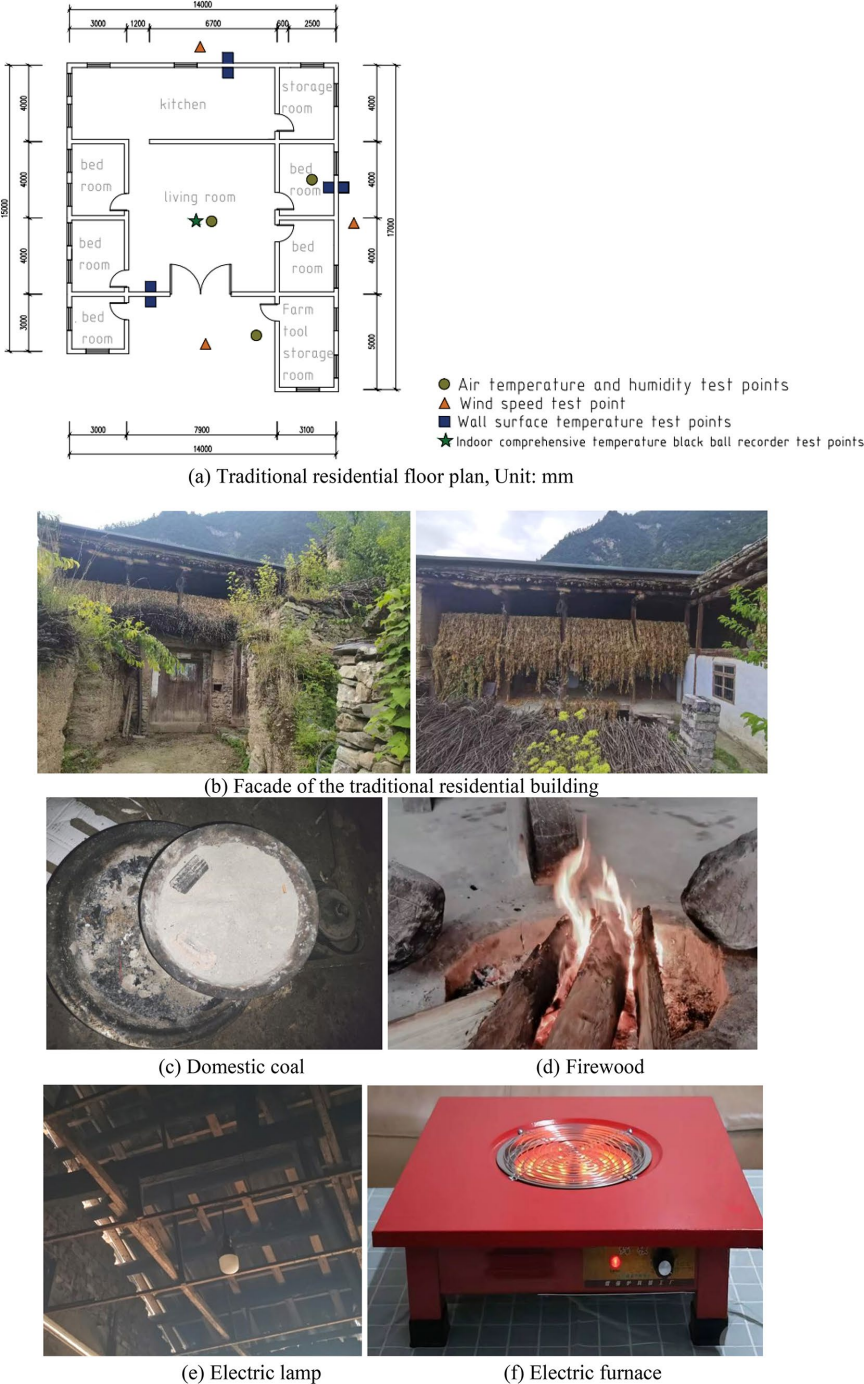
Current situation of traditional dwellings.

Considering external factors that may affect accuracy, we recorded the weather conditions during the test and chose to conduct it under the same conditions, i.e., sunny, light winds (0.6–1.1 m/s), from July 19 to 25, 2024. The temperature and humidity of one of the representative traditional houses is monitored 24 h a day. The selected traditional house was built in 1995; the building plan is “U”-shaped, sitting north and facing south; people live and use a one-story building, as shown in Fig. 4a (the data size unit in the figure is mm), and the overhead space downstairs is used for raising poultry. The building area is 197.7 square meters, the floor height is 2.7 m, sitting north and facing south, the main functional room is composed of a kitchen, hall, bedroom, and storage room, the bathroom is located outside the room, the hall is located in the centre of the building plane, there is a fire pond in the centre of the hall, and people in the plateau area have the life characteristics of “living around the fire” to avoid cold and heat in winter. The main body of the residential envelope is made of local loess masonry, and there are no additional thermal insulation or structural measures. The roof is flat, made of traditional stone and wood structure (Fig. 4b), and covered with loess and Bianma grass, a unique building material in Tibetan areas that is hard, non-perishable, and extremely resistant to severe cold. It is often used on roofs to reflect the characteristics of traditional Tibetan architecture. Different heating and lighting facilities are presented in Fig. 4c–f.

**Fig. 5:**
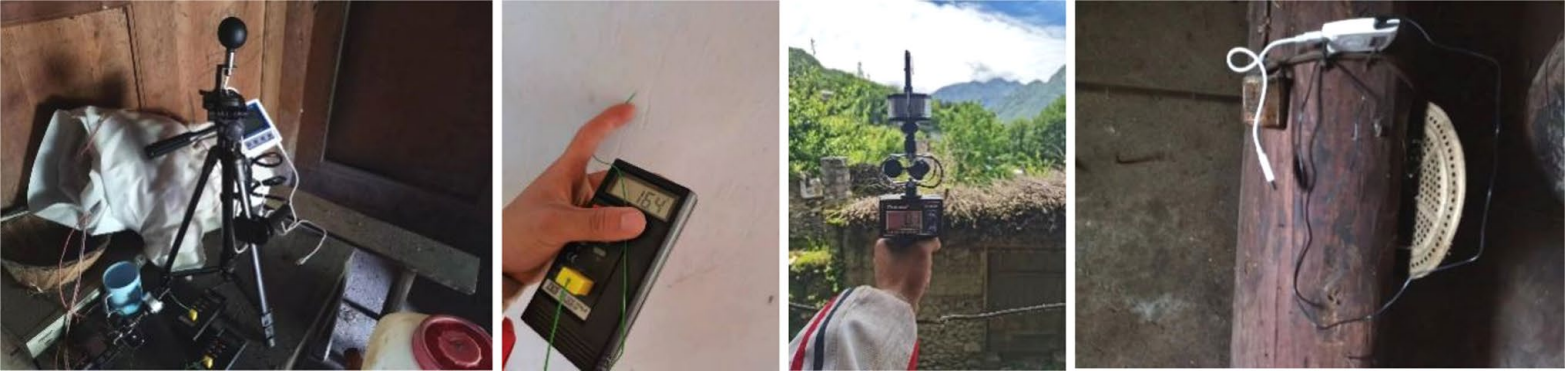
Test instruments.

The measurements are centered on three parameters: the indoor and outdoor air temperature, relative humidity, and wind speed. Before the field test, we calibrated the measuring instruments used to ensure that they can accurately measure the required parameters. The instrument automatically records every 10 min. The indoor temperature and humidity measurement point of the residential house is located at 1.5 m from the ground, and is arranged in the living room and bedroom, which are mainly used in the center of the room, so as to understand the indoor thermal environment of the main use space of the residential house. During the test period, minimize unnecessary activities, maintain the stability of the indoor environment, or maintain a consistent level of human activity, and reduce the impact of human activities on the test site. In order to understand the thermal performance of the envelope structure of the research object, a thermocouple temperature recorder was arranged at the building wall, and the wall temperature measurement points were placed on the inner and outer walls of the south wall of the living room and the bedroom and the inner and outer walls of the east wall of the bedroom for measurement, and the measuring points were still located at 1.5 m from the ground, and the measurements were taken at 6 times a day (6:00, 10:00, 14:00, 18:00, 22:00, 02:00). During the test period, we recorded the ventilation of residential buildings, etc. Indoor and outdoor wind speed is tested with an anemometer, and the wind environment data of 3 points around the target building is continuously monitored: recorded every hour, each measurement time is 1–2 min for each measurement point, and the instantaneous wind speed is recorded every 3–5 s, and 10–15 measured values are obtained each time (variance and average wind speed can be analyzed), and 1–2 dominant wind directions are recorded at each measurement point. The anemometer measurement point is measured six times a day (6:30, 10:30, 14:30, 18:30, 22:30, and 02:30). The instrument distribution is set according to the requirements of JGJ/T132-2009, “Energy Conservation Testing Standard for Residential Buildings”^31^. The specific location of the measurement points is shown in Fig. 4a. The measuring instruments and their related parameters are shown in Tables 2 and Fig. 5.

**Table 2:** Test instruments and parameters.

Instrument name	Specification and type	Measuring range	Measuring accuracy	Test mode
High precision temperature and humidity recorder	RC-4 H	-40–85℃ 0-100%RH	± 0.5℃ ± 3%RH	Automatic/10 min
Hand-held thermocouple temperature recorder	1310TYPE-K	-20-200℃	± 0.2℃	Manual/4 h
Aerovane	Puxicoo/p6-8232	0-360°	± 0.5 directions	Manual/4 h
Blackball temperature recorder	RS-HQ-USB	-40-120℃	± 0.2℃	Automatic/10 min

### Simulation methods

Building energy simulation has become an integral part of the building design process, and through computer simulation, designers can predict the performance of buildings under different conditions, including energy consumption, comfort, environmental impact, etc., and provide data on a series of key indicators for relevant studies, which not only improves the accuracy of the design but also greatly shortens the design cycle and reduces the cost^32^. Among the many simulation tools, Design Builder is favoured for its intuitive operation interface, powerful simulation functions, and wide applicability, which can accurately simulate the heat transfer process of the building envelope, by inputting the thermal conductivity, density, and other parameters of different insulation materials, it can simulate the impact of different building materials on building energy consumption under certain climatic conditions, help designers make optimization decisions in the design stage, and test a variety of design solutions in a virtual environment. The cost of trial and error on the construction site is avoided^33–37^. In addition, Design Builder simulates indoor thermal environments, including temperature, humidity, and air velocity. By simulating the indoor thermal environment under different design scenarios, we can evaluate the impact of various insulation materials and window types on thermal comfort and make recommendations for optimization. For example, by adjusting the size, position, and type of windows, as well as choosing the right insulation, we can achieve better indoor thermal comfort and energy efficiency^38–43^. These research results cover cold regions to tropical and semi-arid regions, from building energy consumption simulation, and indoor thermal environment to new building material applications, as well as passive energy-saving technology, providing strong support for energy conservation, environmental protection, and sustainable development of the building industry. Furthermore, the other authors also compared different simulation tools^44–47^.

### Thermal parameters of the Building envelope and indoor personnel settings

Through on-site measurements and investigations, the traditional houses in this area are found made of local materials, and the basic building structure and materials information of the tested traditional houses is shown in Table 3. With the investigated information, combined with parameter calculation recommendation from the “General Code for Building Energy Conservation and Renewable Energy Utilization” GB55015-2021^48^, and the Thermal Design Code for Civil Buildings GB50176-2016^30^, DesignBuilder (Version 7.0.1.006, https://designbuilder.co.uk), a building energy consumption software, was used to build a digital model of traditional dwellings. The heat transfer resistance of the flat wall of the enclosure structure should be calculated as follows: R0 = Ri + R + Re, (R0 is the heat transfer resistance of the envelope structure; Ri is the heat transfer resistance of the inner surface, which shall be 0.11 m^2^K/W according to the provisions of Appendix B of this Code; The heat exchange resistance of the outer surface of Re shall be 0.04 according to the provisions of Appendix B of this Code), R0 = 0.11 + 0.47 + 0.04 = 0.62 m^2^K/W, and there is a direct relationship between the heat transfer coefficient of the enclosure structure and the heat transfer resistance of the envelope structure, that is: K = 1/R0, so the heat transfer coefficient of the outer wall is K = 1.61 W/m^2^K. Compared with the recommended value of 0.3 W/m^2^K^48^, the heat transfer coefficient of 1.61 W/m^2^K of the exterior walls is obviously high, which will lead to the increase of building energy consumption, which is not conducive to energy conservation and emission reduction. Therefore, it is necessary to reduce the heat transfer coefficient and improve the thermal insulation performance of the building by optimizing the external wall material and adding the insulation layer.

**Table 3:** Thermal parameters of Building envelope.

Constructional element	Structural layer	Thickness /mm	Thermal conductivity (W/m^2^K)	Specific heat capacity (J/kg.K)	Dry Density (kg/m^3^)	Heat transfer coefficient limit in cold region (W/m^2^K)
External wall	Lime gypsum mortar	0.02	0.76	1050	1500	0.3
	Tamping clay	0.50	1.16	1010	2000	
	Lime mortar	0.01	0.81	1050	1600	
Interior wall	Lime mortar	0.01	0.81	1050	1600	1.5
	Tamping clay	0.30	1.16	1010	2000	
	Lime mortar	0.01	0.81	1050	1600	
Roof	Cement mortar	0.02	0.93	1050	1800	0.2
	Plus straw clay	0.20	0.76	1010	1600	
	Cement mortar	0.02	0.93	1050	1800	
	Pine (spruce) board	0.05	0.14	2510	500	
Floor	Cement mortar	0.05	0.93	1050	1800	1.5
	Crushed stone Concrete	0.20	1.51	920	2300	
	Tamping clay	0.30	1.16	1010	2000	
External door	Wood door	0.03	0.21	1400	825	
External window	Wood door common glass	SHGC 0.94		light transmission 0.744		U-Value 1.6

Indoor personnel setting: Considering that the permanent residents of conventional houses are mainly elderly, supplemented by children who usually go home to visit, the model consists of two elderly people and one child to form a family population. The elderly is arranged in the south-facing bedroom on the left side. The living room is a public indoor activity space for the whole family. The total number of people in the family is three: two people in the elderly bedroom, one person in the children’s bedroom, three people in the living room, and the rest of the nobody’s bedroom and bathroom are not considered heat loads. The working and rest time of the staff in the room is combined with the living and rest time of the residents, as shown in Table 4. The lighting equipment is simulated to be turned on at 7 o’clock and turned off at 19 o’clock.

**Table 4:** Personnel presence time period of each hour of a day.

Room types	Time period (h)											
	1 H	2 H	3 H	4 H	5 H	6 H	7 H	8 H	9 H	10 H	11 H	12 H
Bedroom	1.0	1.0	1.0	1.0	1.0	1.0	1.0	0.5	0.5	0.0	0.0	0.0
Living room	0.0	0.0	0.0	0.0	0.0	0.0	0.0	0.5	0.5	1.0	1.0	1.0
Living room (weekends)	0.0	0.0	0.0	0.0	0.0	0.0	0.0	0.0	0.0	0.0	0.0	1.0
	13 H	14 H	15 H	16 H	17 H	18 H	19 H	20 H	21 H	22 H	23 H	24 H
Bedroom	0.0	0.0	0.0	0.0	0.0	0.0	0.0	0.0	0.0	0.5	1.0	1.0
Living room	1.0	1.0	1.0	1.0	1.0	1.0	1.0	1.0	1.0	0.5	0.0	0.0
Living room (weekends)	1.0	1.0	1.0	1.0	1.0	1.0	1.0	1.0	1.0	0.5	0.0	0.0

## Test data and analysis

### Subjective questionnaire analysis

#### Basic information

**Fig. 6:**
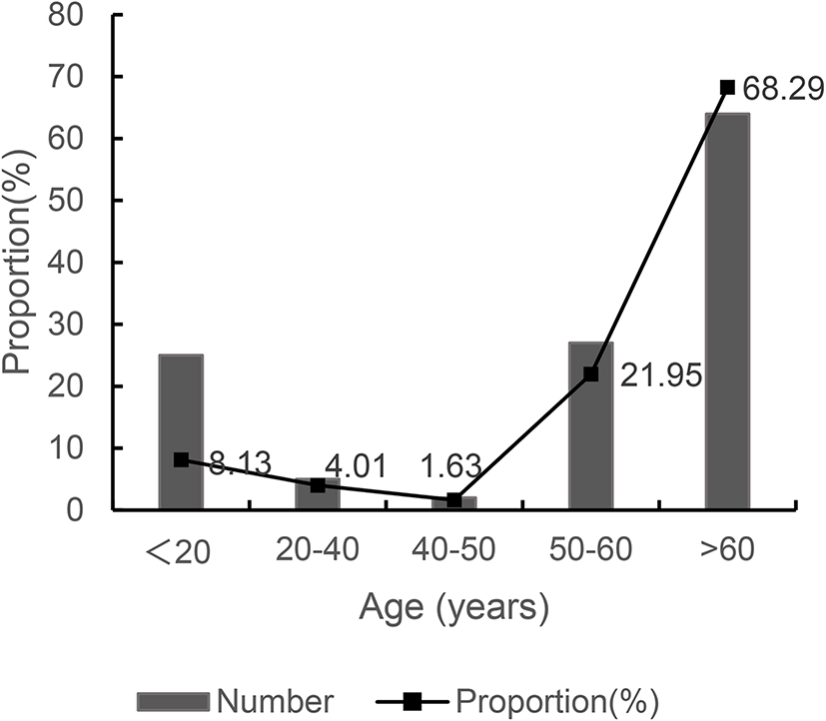
Age structure of the resident population.

Most of the residents who completed the questionnaire were over 60 years old, and the elderly often have deep feelings for the long-term living environment and prefer to live in traditional houses. In contrast, most young and middle-aged people choose to work away from their hometowns and leave their children and the elderly at home. Under the age of 20, the number of people is 25, which is 8.13% of the total number of people; between the ages of 20 and 40, the number of people is 5, which is 4.01%; between the ages of 40 and 50, the number of people is 2, which is 1.63%; between the ages of 50 and 60, the number of people is 27, the proportion is 21.95%; over 60 years old has a number of 64, which takes 68.29%. The respondents in the questionnaire were mainly students and the elderly engaged in manual labor, as shown in Fig. 6. Most of the houses are two-story traditional stone and wood buildings, with a total of about 240 households.

#### Thermal sensation and thermal comfort

At present, the internationally recognized thermal comfort evaluation standard is the Predicted Mean Vote (PMV) proposed by Fanger in 1967^49^, PMV is a subjective thermal perception level evaluation index, which is divided into seven levels according to the human body’s perception of the thermal environment, cold, cool, slightly cool, neutral, slightly warm, warm, and hot, aiming to quantitatively evaluate the thermal comfort of the indoor environment.

**Fig. 7:**
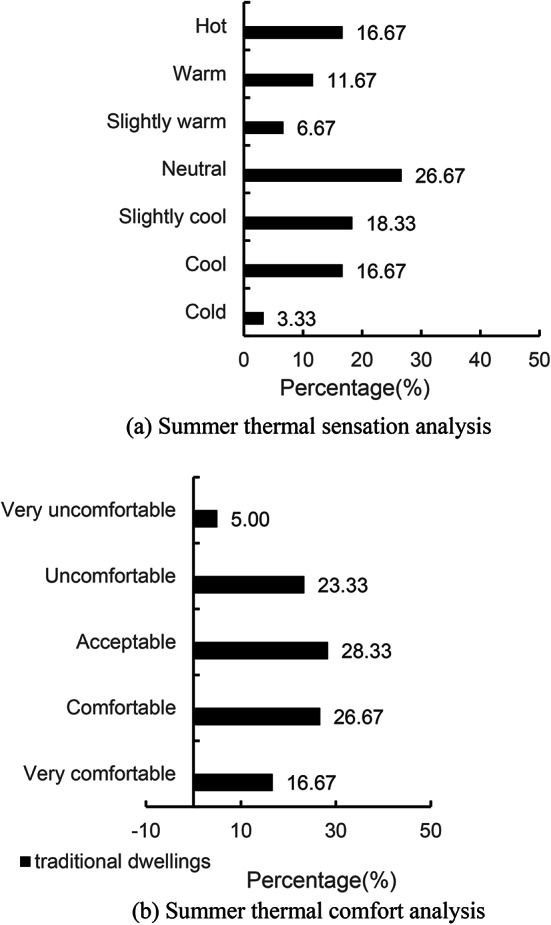
Indoor thermal environment analysis.

The results of the indoor thermal environment survey are shown in Fig. 7, which corroborate the results of our face-to-face interviews with the villagers. Figure 7a shows the residents’ thermal perception evaluation of the indoor thermal environment. Among the traditional houses, 26.67% of the residents felt “moderate”, which means that they felt that the indoor temperature was neither too hot nor too cold, and was in a relatively comfortable range; 18.33% of the residents felt very humid, and “slightly cool”, indicating that some residents felt that the room was somewhat cool, and may even feel slightly cool due to the high humidity; Only 16.67% of residents felt “hot”, which means that most of the indoor environment in Aba Prefecture does not make residents feel too hot in summer. It can be concluded that most people in Aba Prefecture feel moderate and not hot in summer; As can be seen in Fig. 7b and 28.33% of residents feel “acceptable”, which is the highest proportion of options, indicating that most residents are relatively satisfied with the current indoor thermal environment. The proportion of people who felt uncomfortable indoors was 23.33%, which may be related to various factors such as indoor temperature, humidity, and wind speed. 26.67% of residents feel “comfortable”, which is like the proportion of “acceptable” residents, which further illustrates the satisfaction of the majority of residents with the indoor thermal environment. In addition, 51.67% of the residents hope that the wind speed will be stronger, which indicates that many residents feel that the indoor airflow is not enough, and hope to increase the wind speed to improve comfort. 63.33% of residents felt that the room was damp and needed to be dehumidified. This is a relatively high percentage, indicating that dehumidification is one of the issues that needs to be paid attention to in Aba Prefecture.

On the whole, young residents are generally more sensitive to temperature and prefer a cooler environment, and this part of the residents has a higher evaluation of “moderate” or “slightly cool”. Young people are more active have a fast metabolism and are more inclined to have stronger air flow to improve comfort, and the proportion of residents who want stronger wind speed may be higher. Young people may have a relatively low tolerance for humidity and are more likely to experience damp discomfort. As a result, there may also be a higher proportion of residents who feel that their rooms need to be dehumidified, and although there are people of all ages who feel uncomfortable with dampness, older residents may be more susceptible to humidity due to decreased physical function. There is a certain relationship between the demographic characteristics of residents and the preference for indoor thermal environment, and these analysis results provide a useful reference for optimal design.

### Summer thermal environment data analysis

#### Indoor and outdoor air temperature

During the test period, the average outdoor temperature is 12.3 °C, the highest temperature is 17.8 °C, usually around 15:00 a day, and the lowest temperature is 9.3 °C, which usually occurs around 5:00, and the temperature difference reaches 8.5 °C; The relative humidity of the air varies from 53.5 to 67.4%, while the average relative humidity is 62.7%. The outdoor temperature changes with the change of solar radiation, and the increase of solar radiation leads to an increase in outdoor temperature and a decrease in the relative humidity of the air, while a decrease in solar radiation leads to a decrease in the outdoor temperature and an increase in the relative humidity of the air.

**Fig. 8:**
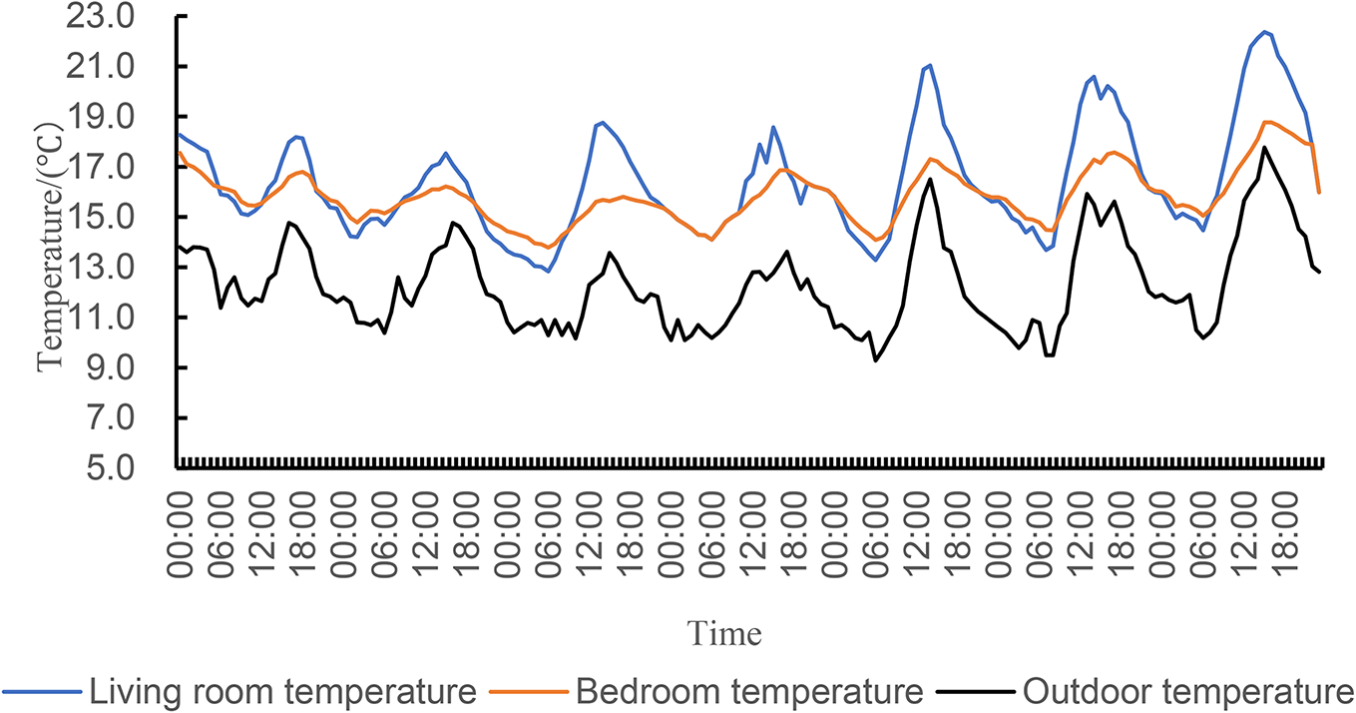
Indoor and outdoor temperature of traditional dwellings.

Figure 8; Table 5 show the analysis of temperature data in the living room and bedroom. During the experiment, the average temperature in the living room and bedroom of the traditional house was 16.4 °C and 15.8 °C, respectively, with fluctuations ranging from 12.8 to 22.4 °C and 13.8–18.8 °C. The highest temperature in the living room is 22.4 °C, which occurs at about 16:00, and the lowest temperature is 12.8 °C, which occurs at about 6:00–7:00. The test bedroom had a temperature difference of 5 °C throughout the day, with a maximum temperature of 18.8 °C occurring at approximately 16:00 and a minimum of 13.8 °C occurring between 5:00 and 7:00. The comparison shows that the indoor and outdoor air changes are basically synchronized, and there is a delay of 1–2 h between the maximum and minimum temperature of the outdoor and indoor air, indicating that the thermal resistance and thermal inertia of the traditional residential walls are larger, and they have better thermal insulation performance.

**Table 5:** Indoor and outdoor temperature statistics of traditional dwellings in summer season.

Position	Mean temperature/℃	Maximum temperature/℃	Minimum temperature/℃	Temperature variation/℃
Outdoor	12.3	17.8	9.3	8.5
Living room	16.4	22.4	12.8	9.6
Bedroom	15.8	18.8	13.8	5.0

#### Indoor and outdoor air relative humidity

**Fig. 9:**
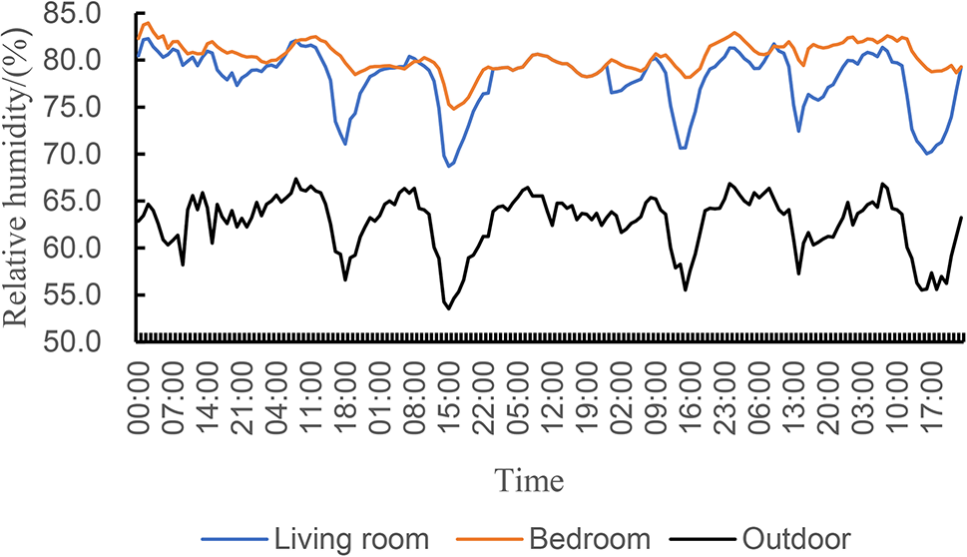
Indoor and outdoor air relative humidity of traditional houses.

Figure 9; Table 6 show the analysis of the relative humidity data of the study subjects. During the test period, the average humidity of the living room and bedroom of traditional houses was 78.0% and 80.3%, respectively, which were higher than the requirement of 60% average indoor humidity in summer in China’s “Evaluation Standard for Indoor Thermal Environment of Civil Buildings” (GB/T50785-2012)^50^. The relative humidity fluctuates from 68.7%~82.3% in the living room and 74.8-84% in the bedroom. The outdoor average humidity of 62.7% is significantly lower than the indoor relative humidity, and the outdoor non-rainy day, the natural ventilation and lighting are good, and the water will be taken away and dispersed in time after volatilization, so its relative humidity is lower than that indoors, which also shows that the effective organization of natural ventilation in the layout of the building space can also reduce the relative humidity of indoor air, which is an effective strategy to improve the indoor comfort of traditional houses. In addition, the interior floor of traditional houses does not have a moisture barrier, and the moisture in the foundation soil penetrates upward, resulting in high indoor humidity^51–54^. In the following simulation design of this paper, effective optimization measures will be taken to solve the above problems.

**Table 6:** Indoor and outdoor relative humidity statistics of traditional dwellings in summer season.

Position	Medial humidity/%	Maximum humidity/%	Minimum humidity/%	Moisture variation/%
Outdoor	62.7	67.4	53.5	13.9
Living room	78.0	82.3	68.7	13.6
Bedroom	80.3	84.0	74.8	9.2

#### Wall thermal performance

The south wall of the tested living room and the wall surface of the bedroom were studied, and the temperature of the outer wall surface was always higher than that of the inner wall surface during the measurement period, and the temperature of the inner wall surface changed with the change of the temperature of the outer wall surface, and there was a strong correlation between the two, which confirmed that the temperature of the outer wall surface was one of the important factors affecting the temperature of the inner wall surface. The highest outer wall temperature was 21.6 °C on the south wall of the living room, with a peak of 21.6 °C at 2 p.m. on July 25, and the temperature of the inner wall was 19.4 °C, with a temperature difference of 2.2 °C, and the highest outer wall temperature of 20.8 °C in the bedroom occurred at 2 p.m. on July 23, with a maximum temperature of 19.6 °C and a temperature difference of 1.2 °C. It shows that the temperature difference between the outer wall and the inner wall of the south wall of the living room is more obvious than that of the bedroom due to the orientation and direct sunlight, as shown in Fig. 9. Through the study of the temperature of the south wall of the living room and the inner and outer walls of the bedroom wall, we can better understand the behavior of the building’s thermal environment and propose corresponding optimization measures to improve living comfort.

**Fig. 10:**
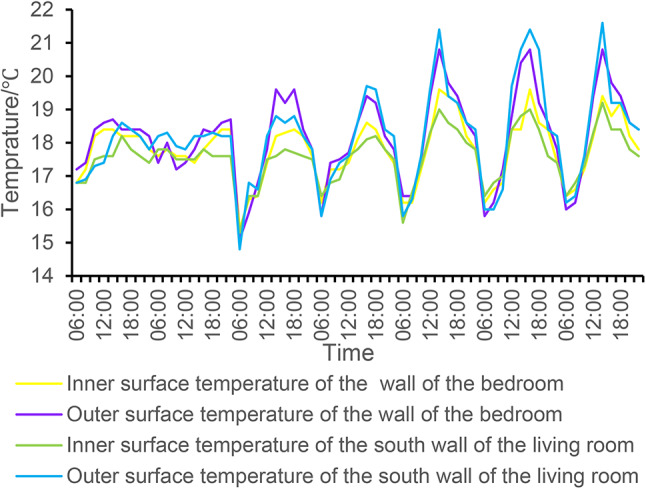
Temperature analysis of the interior and exterior walls of the living room and bedroom.

## Comparative analysis of simulated data and measured data

**Fig. 11:**
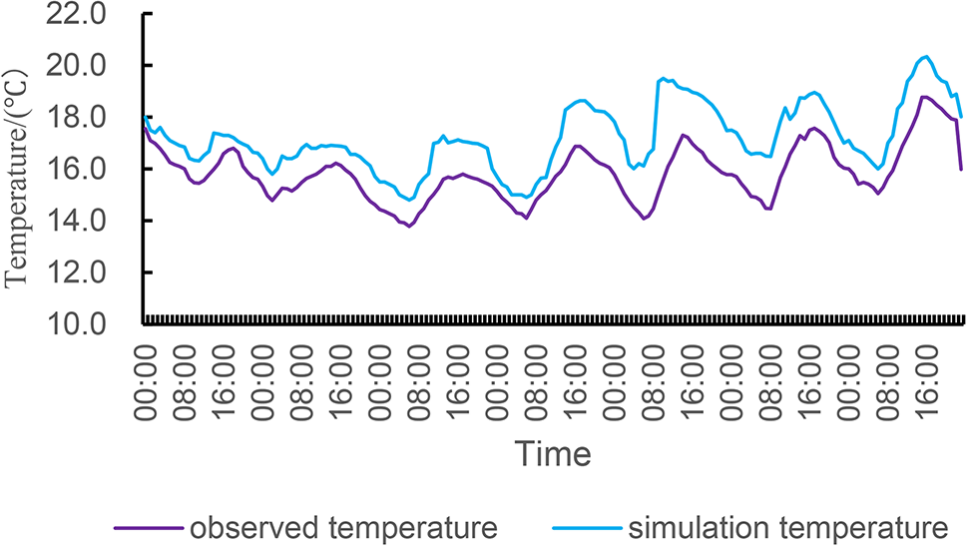
The measured temperature and simulated temperature of the bedroom.

**Fig. 12:**
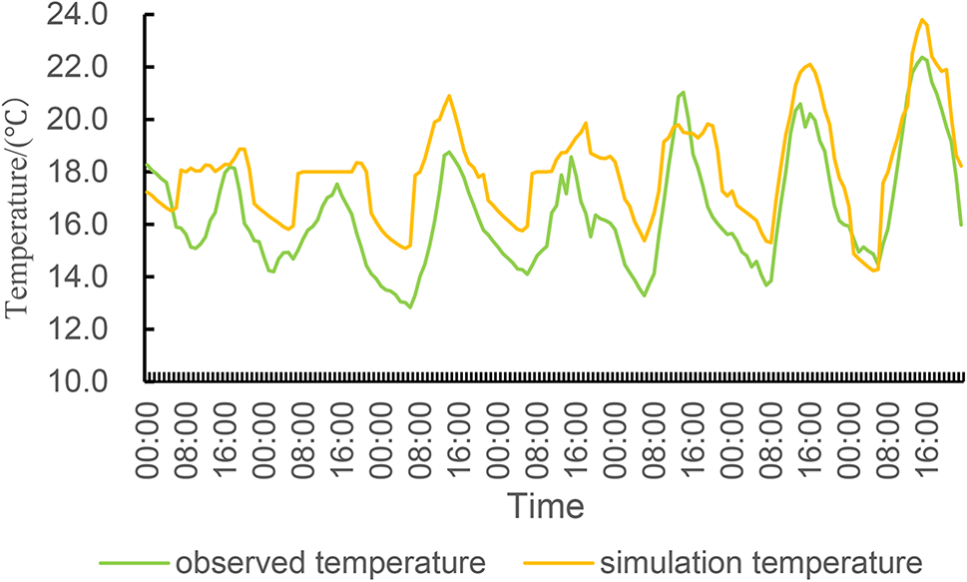
The measured temperature and simulated temperature of the living room.

In order to verify the suitability of the simulated boundary conditions and the software settings, the indoor temperature conditions of the traditional residential houses are simulated, as shown in Figs. 11 and 12 are the comparison curves of the indoor temperature simulation data and the measured data of the bedroom and living room respectively, and the temperature change trend of the two is roughly the same, the temperature is lower in the morning, gradually increases with the enhancement of sunshine, and gradually decreases after the evening, so this can preliminarily show that the simulated boundary conditions and software settings can capture the basic thermal dynamic process, and the data difference is small, It shows that the simulation results are more accurate, and it can be seen that the thermal parameters and boundary conditions of the model are set reasonably, and it can also be determined that the application of DesignBuilder in the study of building thermal performance in the high altitude area of western Sichuan is reasonable and suitable, which can accurately simulate and analyse the thermal performance of buildings in extreme environments, and provide strong technical support for the optimization design and energy-saving renovation of traditional residential buildings in the future.

At the same time, statistical indicators such as RMSE (Root Mean Square Error) and MAPE (Mean Absolute Percentage Error) are used to verify the accuracy of the simulation model. RMSE is a measure of the difference between the simulated and measured values and is calculated as follows:

$$\:\text{R}\text{M}\text{S}\text{E}=\sqrt{\frac{1}{n}\sum\limits_{i=1}^{n}\:({y}_{i}-{\widehat{y}}_{i}{)}^{2}}$$

n is the number of data points; $\:{y}_{i}$ is the ith measured value; $\:{\widehat{y}}_{i}$ is the ith simulated value;

MAPE is a measure of the degree of deviation of the simulated value from the measured value, and is calculated as follows:

$$\:\text{M}\text{A}\text{P}\text{E}=\frac{1}{n}\sum\limits_{i=1}^{n}\:\left|\frac{{y}_{i}-{\widehat{y}}_{i}}{{y}_{i}}\right|\times\:100\text{\%}$$

n is the number of data points; $\:{y}_{i}$ is the ith measured value; $\:{\widehat{y}}_{i}$ is the ith simulated value.

According to the dataset of measured temperature and simulated temperature in the bedroom and the above formulas, the RMSE is calculated: 1.49, MAPE: 8.62%; According to the dataset of measured temperature and simulated temperature in the living room and the above formula, the RMSE is calculated: 1.96, and MAPE: 11.13%. These metrics can be used to assess the degree of fit between the simulated and measured data. The smaller the RMSE value, the smaller the difference between the simulated data and the measured data; The lower the MAPE value, the higher the accuracy of the simulated data.

## Simulation optimization of traditional dwellings

### Parameter optimization and simulation of Building envelope structure

Using the typical meteorological data of Aba Prefecture in the Chinese Standard Weather Data (CSWD) of DesignBuilder, the indoor thermal environment of the building was simulated in July of the test period; in the meantime, The indoor temperature is set to 18 °C. Except for the bedroom and living room where people live, the rest of the room does not require a heat load. According to commonly used building materials combined with the General Code for Building Energy Conservation and Renewable Energy Utilization GB55015-2021, when the building is no more than 3 floors, the heat transfer coefficient limit for roof is 0.2 W/(m^2^·K), for exterior wall is 0.3 W/(m^2^·K), for interior wall is 1.5 W/(m^2^·K), and for floor is 1.5 W/(m^2^·K). Combined with the calculation parameters of the thermal physical properties of building materials specified in the “Code for thermal design of civil buildings” GB50176–2016. The main materials of the optimized simulated envelope structure, and their thermal conductivity, specific heat capacity, dry density, and other interrelating parameters were determined. The thermal parameters of external walls, floor and roof in the software are given in the Tables 7, 8 and 9.

**Table 7:** Thermal parameters of external walls.

Materials and parameters	Thickness (mm)	Thermal conductivity (W/m^2^·K)	Specific heat capacity (J/kg·K)	Dry density (kg/m^3^)	Thermal resistance (m^2^·K/W)
Cement mortar	30	0.93	1050	1800	0.03
Extruded polystyrene sheet (XPS)	120	0.03	1380	35	4
Aerated concrete	400	0.14	1050	500	2.86
Cement mortar	20	0.93	1050	1800	0.02
Total thermal resistance			7.06 m^2^·K/W		
Heat transfer coefficient			0.142 W/(m^2^·K)		

**Table 8:** Thermal parameters of floor.

Materials and Parameters	Thickness (mm)	Thermal conductivity (W/m^2^·K)	Specific heat capacity (J/kg·K)	Dry density (kg/m³)	Thermal resistance (m^2^·K/W)
Cement mortar	20	0.93	1050	1800	0.02
Asphalt malthoid	50	0.17	1470	600	0.29
Cement mortar	20	0.93	1050	1800	0.02
Crushed stone concrete	150	1.51	920	2300	0.10
Total thermal resistance			0.58 m^2^·K/W		
Heat transfer coefficient			1.724 W/(m^2^·K)		

**Table 9:** Thermal parameters of roof.

Materials and parameters	Thickness (mm)	Thermal conductivity (W/m^2^·K)	Specific heat capacity (J/kg·K)	Dry density (kg/m^3^)	Thermal resistance (m^2^·K/W)
Cement mortar	20	0.93	1050	1800	0.02
Asphalt malthoid	50	0.17	1470	600	0.29
Extruded polystyrene sheet (XPS)	180	0.03	1380	35	6
Cement mortar	20	0.93	1050	1800	0.02
Reinforced concrete	150	1.74	920	2500	0.09
Total thermal resistance			6.57 m^2^·K/W		
Heat transfer coefficient			0.152 W/(m^2^·K)		

### Building plane function optimization

**Fig. 13:**
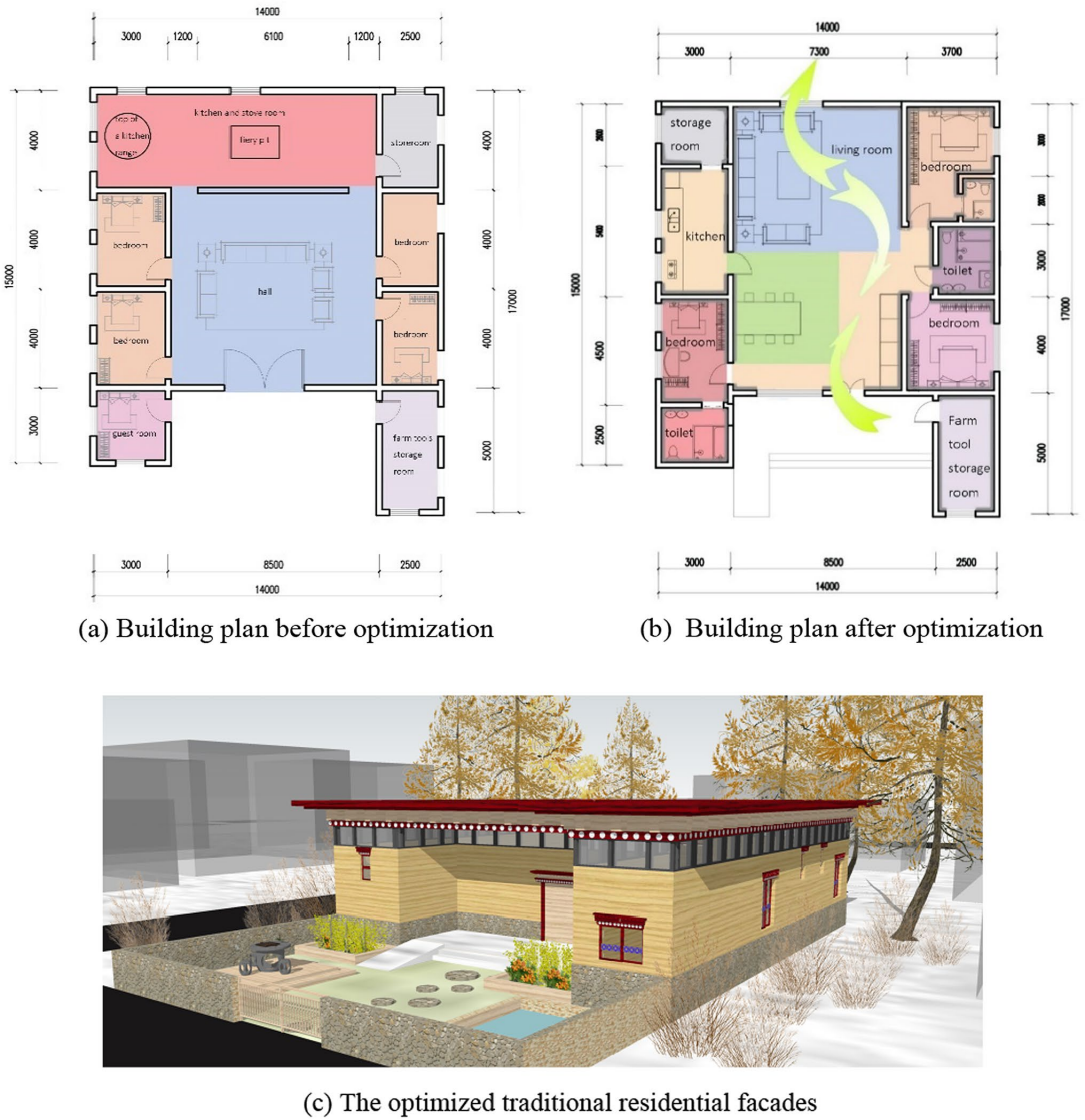
Comparison of traditional dwellings before and after optimization.

The existing traditional houses have poor ventilation and lighting, and the indoor relative humidity is high. The architectural design can be explained in Fig. 13a: the entrance door of the living room and the adjacent room do not form a ventilation hall. Secondly, there is a kitchen at the back of the living room, and there is no external wall of the living room with direct windows, resulting in poor indoor ventilation and poor air circulation, and even if the moisture is volatilized, it will remain indoors, resulting in high indoor relative humidity, so indoor ventilation and dehumidification treatment should be carried out to obtain better indoor thermal comfort; In addition, there is no moisture-proof structural layer on the indoor floor of the traditional house, and the moisture in the foundation soil penetrates upward, resulting in high indoor humidity, and the outer envelope structure such as the exterior wall and roof does not have a waterproof structural layer, resulting in the retention of rainwater and so on, resulting in a high relative humidity of the house, so the waterproof and moisture-proof structural layer should also be done in the enclosure structure part, so as to effectively eliminate the phenomenon of ground moisture return and wall mildew in the traditional house.

The optimized architectural plan design is shown in Fig. 13b, the living room space is expanded, the original kitchen on the north side is adjusted to the west side, the south-facing windows are added, the south window-to-wall ratio is increased, and the north windows of the living room form a penetrating air, which improves air circulation, improves ventilation and lighting, and at the same time considers the local cold climate conditions, and reduces the window opening area on the north, east and west sides; Before the renovation, people had to pass through the living room to enter and exit the bedroom, kitchen and other rooms, resulting in mutual interference between flow lines and line of sight. After the optimization, the functional zoning of the building plan is clear, and the indoor toilet that is not available in the existing traditional houses is added to prevent residents from going to the toilet outdoors in the cold winter. The bedroom, kitchen, bathroom, and other private and functional spaces are separated from the living room and other public activity spaces, and the indoor circulation line is re-planned to make the activities of the personnel between different rooms smoother and more efficient, thereby avoiding streamline crossover and line of sight interference. The reformed plan ensures that each room has good natural lighting and ventilation. The indoor environment was improved by adjusting the position and size of windows, separating the roof and wall, and adding side windows, as shown in Fig. 13c. The façade of the optimized traditional houses is simple and rugged, retaining the unique architectural style and cultural connotation of the local ethnic minorities, and the plan focuses on improving the independence and efficiency of the space while maintaining visual comfort and privacy.

### Potential implementation challenges and solutions

While these optimization measures are technically possible, they may still face a variety of potential economic, cultural, technological, and social challenges in practice.

Economic challenges: for example, adding a waterproof and moisture-resistant structural layer to the envelope requires a corresponding financial investment, which can be an unbearable burden for residents with limited financial resources. It is necessary to actively apply for national poverty alleviation funds or local governments can set up special funds to support the implementation of these optimization measures. At the same time, the participation of private enterprises and social organizations should be encouraged to reduce the economic pressure on residents.

Cultural challenges: optimization measures may not be in harmony with the architectural style of traditional houses, which requires that the architectural style and cultural characteristics of local traditional houses be fully considered when designing optimization measures to ensure that the optimized houses maintain the local architectural style and maintain harmony with the surrounding environment.

Technical challenges: in the traditional houses of ethnic minorities, the construction difficulties brought about by the above-mentioned optimization measures may be greater, relying on the experience left by local craftsmen, villagers and ancestors, traditional customs and culture, and personal intuition, and the lack of scientific and standardized professional knowledge, which can easily lead to potential safety hazards and quality problems. A professional construction team should be introduced to use advanced technical means to deal with complex construction problems. At the same time, communicate closely with residents to understand their needs and expectations to ensure a smooth construction process.

Social challenges: some residents may be sceptical of the optimization measures and are unwilling to cooperate with the implementation, so they can understand the needs and concerns of residents through community meetings, questionnaires, etc., and provide detailed answers and explanations. At the same time, residents who have already implemented the optimization measures are invited to share their experiences and feelings to enhance the trust and cooperation of other residents.

### Simulation results and analysis before and after optimization

**Fig. 14:**
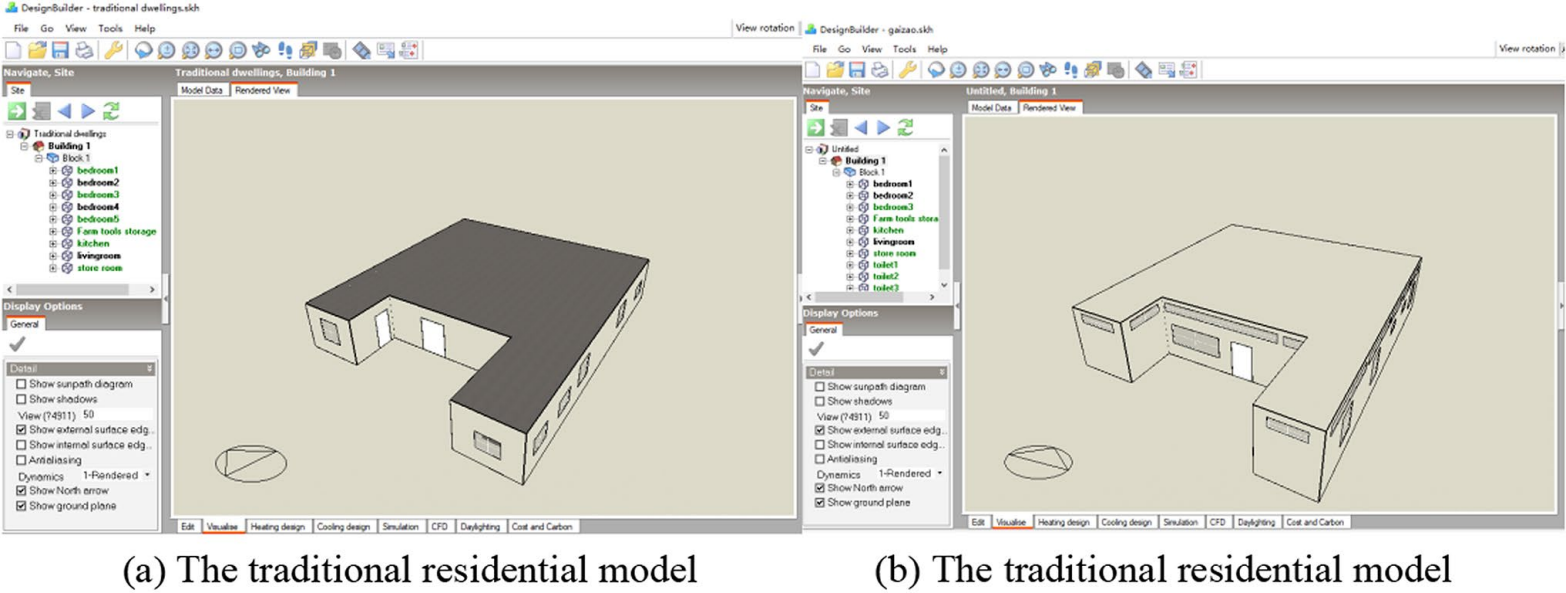
Traditional residential model.

The simulation time is selected from July 19 to 25, 2024 for summer and the winter heating period is from October 1, 2024 to March 31, 2025. The model is shown in Fig. 14, and the indoor thermal environment and winter energy consumption of traditional dwellings before and after the transformation and optimization are compared and analysed.

**Fig. 15:**
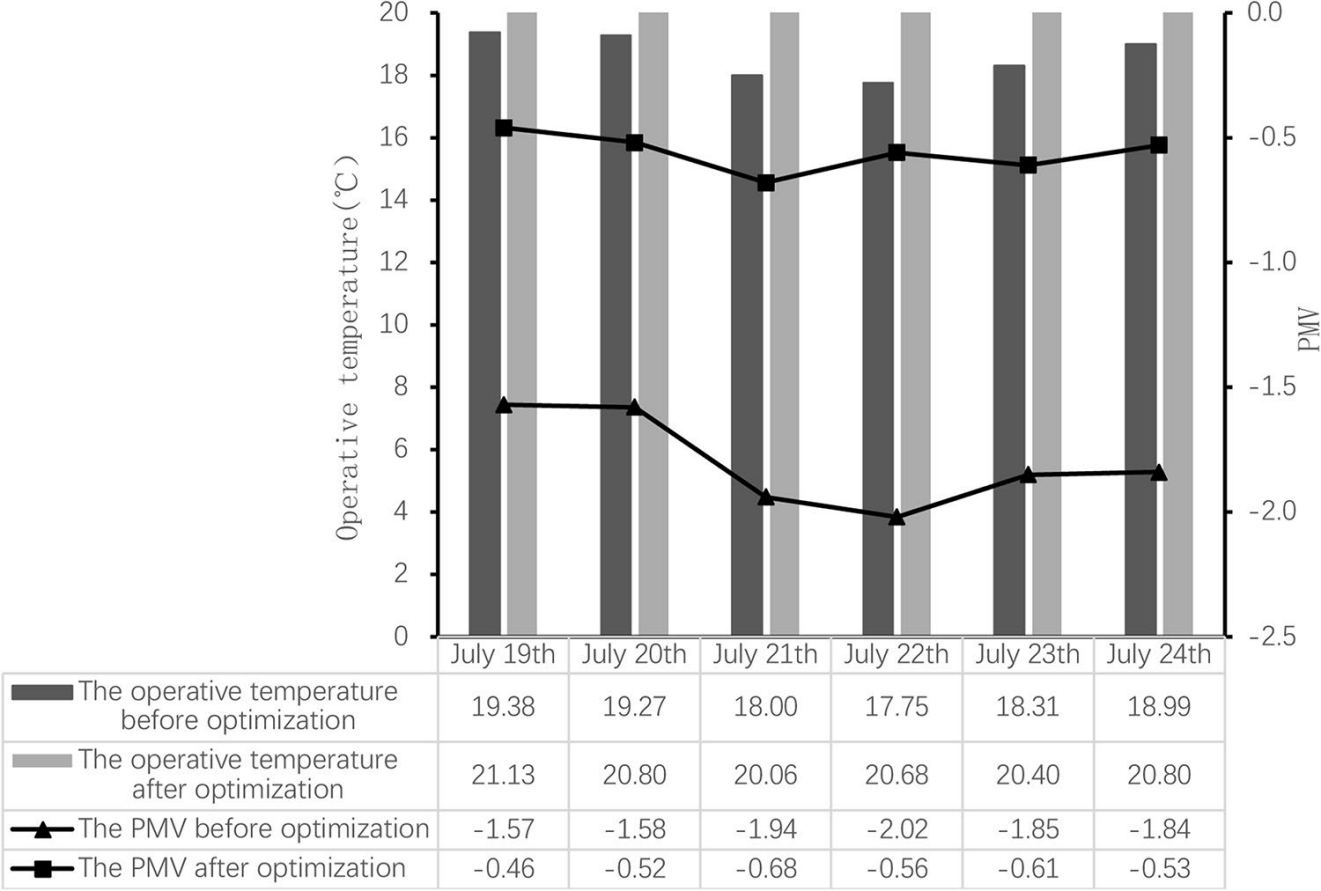
Comparison of indoor thermal environment simulation before and after optimization of traditional dwellings.

When simulating the indoor thermal environment, on the simulation output interface, turn off the Heating option to simulate the living room, as shown in Fig. 15. As can be seen from the figure, the optimized enclosure structure enhances the thermal insulation performance, uses materials with low thermal conductivity, etc., reduces the loss of indoor heat, and the indoor temperature of the living room increases every day, and the indoor temperature can be maintained at a high level throughout the day. This effect is especially noticeable in winter, and in other seasons it can also improve living comfort by reducing the temperature difference between day and night. The optimization of the envelope structure is also accompanied by the addition of a waterproof and moisture-proof building layer, which reduces the indoor humidity and combines with the increase in indoor temperature, which is an important reason for the significant increase in the PMV value of the living room. Higher PMV values mean that residents can enjoy a more comfortable indoor environment, contributing to a higher quality of life and satisfaction. The results of this simulation show that by optimizing the envelope, the living room shows a positive effect of increasing the indoor temperature and PMV value in the simulation. These changes not only improve the comfort and quality of occupancy but also help reduce energy consumption and promote sustainable development.

**Fig. 16:**
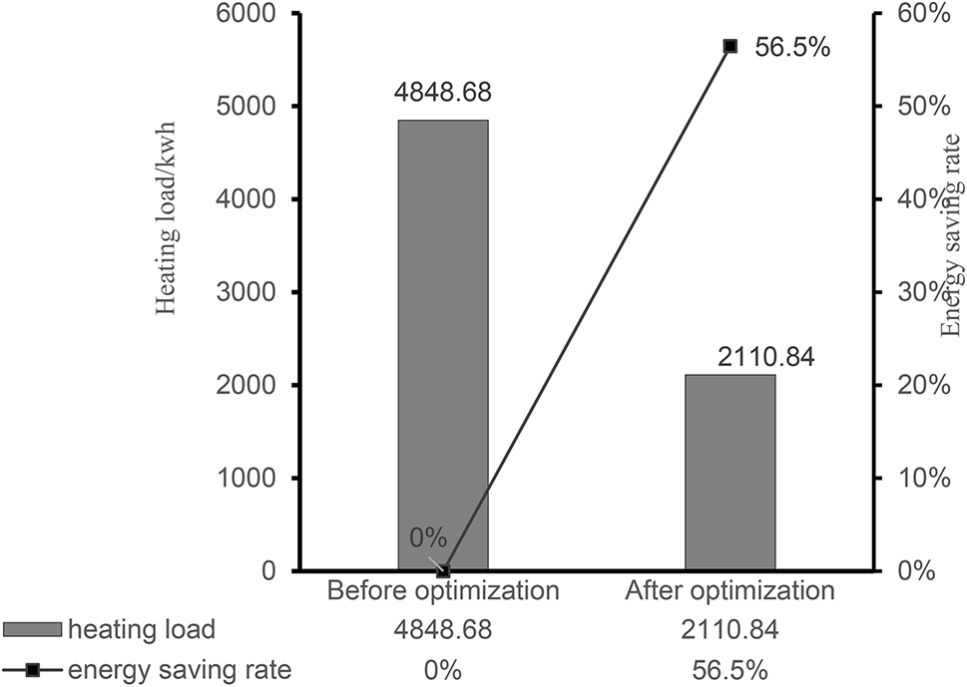
Simulation comparison of heating load in winter before and after optimization of traditional dwellings.

**Fig. 17:**
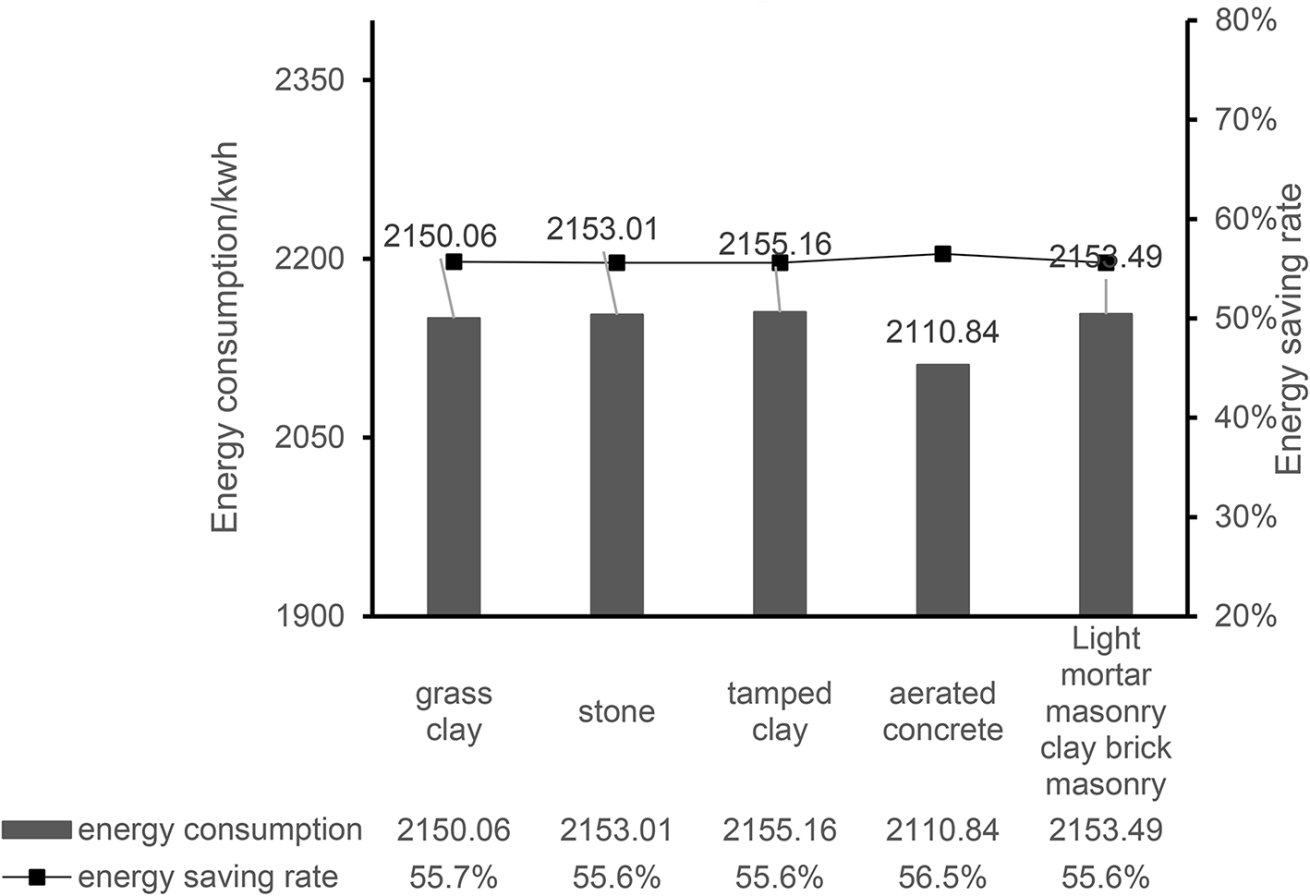
Energy consumption and energy saving rate of different exterior wall structure materials.

Simulate the energy consumption of the entire building based on winter heating, as shown in Fig. 16. As can be seen from the figure, the heat load in the test period of the model before optimization is 4848.68 kWh, and the maximum heat load occurs in December 2024 and January 2025, which are 1014.93 kWh and 1016.21 kWh, respectively. In the optimized building model, the material changes of the main structure of the exterior wall are simulated and compared, and the thermal parameters of the exterior wall are shown in Table 10: when the aerated concrete material is used, the heat load in the model test period is 2110.84 kW·h, which is compared with the heat load of 4848.68 kW·h before optimization, which significantly reduces the heating energy consumption in winter and saves about 56.5%. When the grass clay is used as the enclosure structure material, the heat load during the model test period is 2150.06 kW·h, and the energy saving is about 55.7%. When stone is used, the heat load during the model test period is 2153.01 kW·h, and the energy saving is about 55.6%. When the tamped clay material is used, the heat load during the model test period is 2155.16 kW·h, and the energy saving is about 55.6%. When light mortar masonry is used to build clay brick masonry, the heat load during the model test period is 2153.49 kW·h, and the energy saving is about 55.6%. The use of different exterior wall main structural materials significantly reduced the heat load during the model test period, and the energy saving rate generally remained at a high level (about 55.6–56.5%), as shown in Fig. 17. This shows that the optimization measures show good robustness under a variety of conditions, which provides strong support for practical engineering applications.

**Table 10:** Thermal parameters of exterior wall materials.

Materials and parameters	Thickness d/mm	Thermal conductivity λ /(W·m^− 1^·K^− 1^)	Specific heat capacity c /(J·kg^− 1^·K^− 1^)	Dry density ρ /(kg·m^− 3^)	Thermal resistance *R* /(m^2^·K·W^− 1^)
Grass clay	400	0.58	1010	1 400	0.69
Stone	400	2.04	920	2 400	0.20
Aerated concrete	400	0.14	1 050	500	2.86
Tamped clay	400	0.93	1 010	1 800	0.43
Light mortar masonry clay brick masonry	400	0.76	1 050	1 700	0.53

### Building orientation

Aba Prefecture is in the western Sichuan Plateau, with less cloud cover and strong solar radiation. The building orientation has a significant impact on the amount of solar radiation received. The orientation of the building model is simulated every 15° from October 1 of 2024 to March 31 of 2025.

**Fig. 18:**
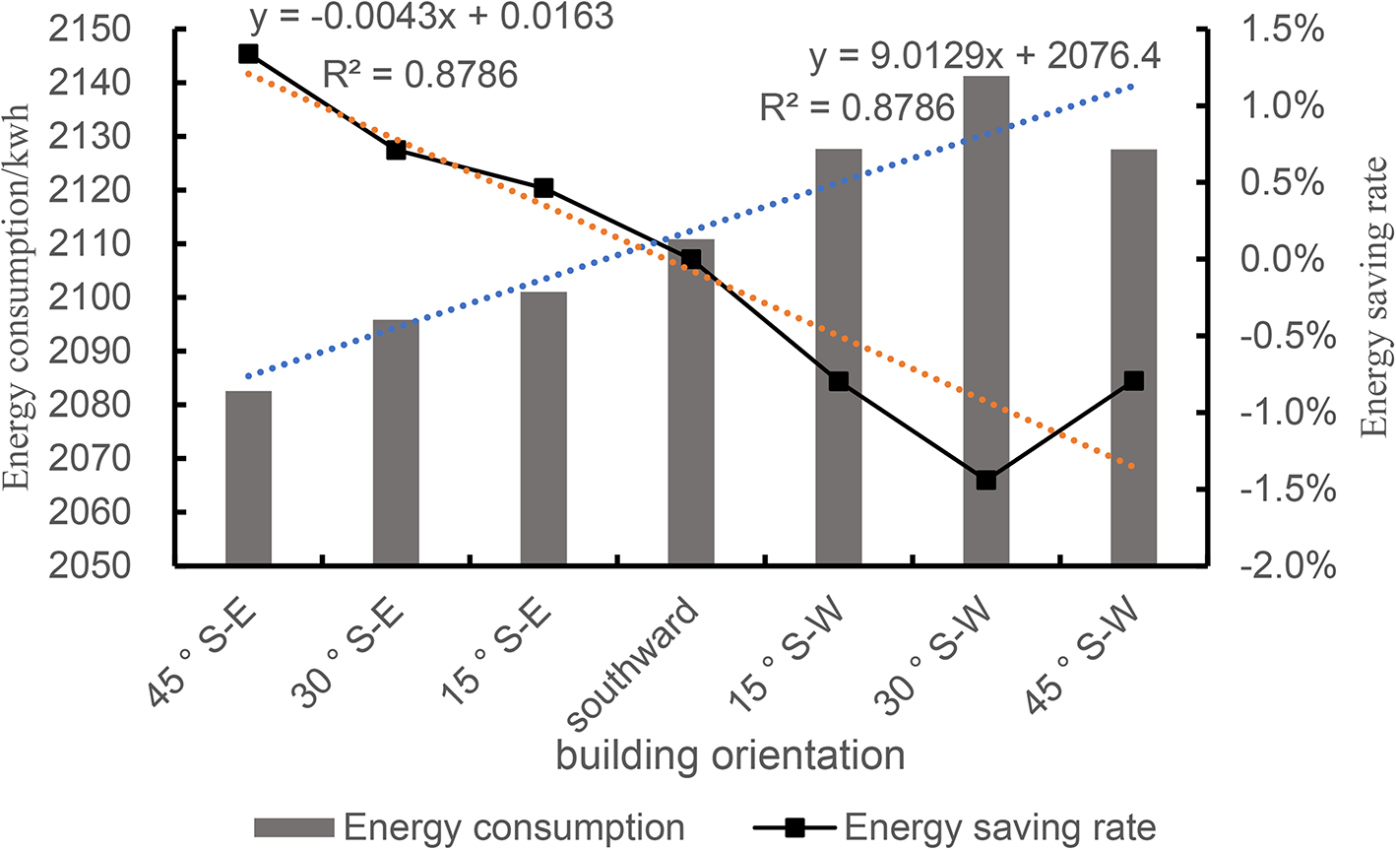
Simulation data of energy consumption for different building orientations.

The relationship between building orientation and building energy consumption is as follows: when the building orientation is south-west, the heating energy consumption is relatively large, and with the gradual shift of the orientation to the east, the heating energy consumption gradually decreases, and the heating energy consumption reaches a relatively low at 45° south-east. From this angle, the building receives more direct and abundant sunlight for most of the day, which increases the natural temperature of the interior. Therefore, based on the complex relationship between building orientation and solar radiation, indoor thermal environment, and heating energy consumption, the local housing design should try to avoid orientations that are not conducive to energy conservation, such as west orientation. By collating all the simulated data, the relationship between orientation and winter energy consumption can be obtained, and the quantitative relationship can be obtained by linear regression, as shown in Fig. 18.

### Analysis of CO_2_ emissions and economic suitability of heating during Building operation

#### Cost comparison

- Heating methods with electric tools. When calculating the energy saving rate of the envelope structure according to the “Green Performance Calculation Standard for Civil Buildings” JGJ / T449-2018^55^, the annual heating energy consumption during the building operation period of the building should be calculated according to the following formula:

$${{\text{E}}_{h,bld}}=\frac{{{{\text{Q}}_{H,bld}}}}{{{\theta _1}}}$$

Where *E*_H, bld_ is the annual heating energy consumption of the building; *Q*_H, bld_ is the cumulative heat consumption of the building throughout the year, which is determined by simulation calculation; *θ*_1_ is the conversion weight of the comprehensive efficiency of the heating system. According to the optimized simulation calculation, *Q*_H, bld_ =2110.84 kW·h, with the corresponding *θ*_1_ = 1.6, then *E*_H, bld_ =1319.28 kw·h. According to the “Building Carbon Emission Calculation Standard” GB/T51366-2019^56^, Sichuan Province belongs to the Central China regional power grid, and the carbon emission factor f = 0.5257 kg/(kw·h), so the CO_2_ emissions of heating during operation are *E*_H, bld_ × f = 1319.28 × 0.5257 = 693.55 kg. Finally, the electricity fee is calculated according to the electricity price, the price of 1 kW·h electricity is between 0.56 ~ 0.62 yuan, calculated at 0.6 yuan/(kw·h), then the annual electricity fee during the operation period C_annual_ = *E*_H, bld_× electricity price = 1319.28 × 0.6 = 791.57 yuan.

(b) Heating methods with household coal. According to the “General Code for Building Energy Conservation and Renewable Energy Utilization” GB55015-2021, the converted weight of the comprehensive efficiency of the heating system is *θ*_1_ = 0.88. According to the above formula and simulation calculation, *E*_*H, bld*_ = 2398.68 kw·h. The CO2 emission factor per unit calorific value of anthracite coal is 94.44 t/TJ, 1 kW·h = 3.6 × 106 J, 1 TJ = 1012 J, so the carbon emission is 815.51 kg. The coal burning volume of 1 kw·h is equal to 1.229 × 10^− 4^ t, the coal burning volume during the operation period is 0.29 t, the bituminous coal price is calculated at 510 yuan/t, and the annual coal burning fee during the operation period is 147.9 yuan.
(c) Heating methods with biomass (wood, hay, etc.). According to the General Code for Building Energy Conservation and Renewable Energy Utilization GB55015-2021, the converted weight of the comprehensive efficiency of the heating system is 0.8. According to the simulation results and the above formula, E_H, bld_=2638.55kw·h, combined with the “Building Carbon Emission Calculation Standard” GB/T51366-2019, the effective CO2 emission factor of wood is 95 t/TJ, so the carbon emission is 902.38 kg. The amount of wood used in 1 kW·h is equal to 4.0 × 10^− 4^ t, the price of wood is calculated at 839.44 yuan/t, and the timber cost is 889.8 yuan during the annual operation period.

**Fig. 19:**
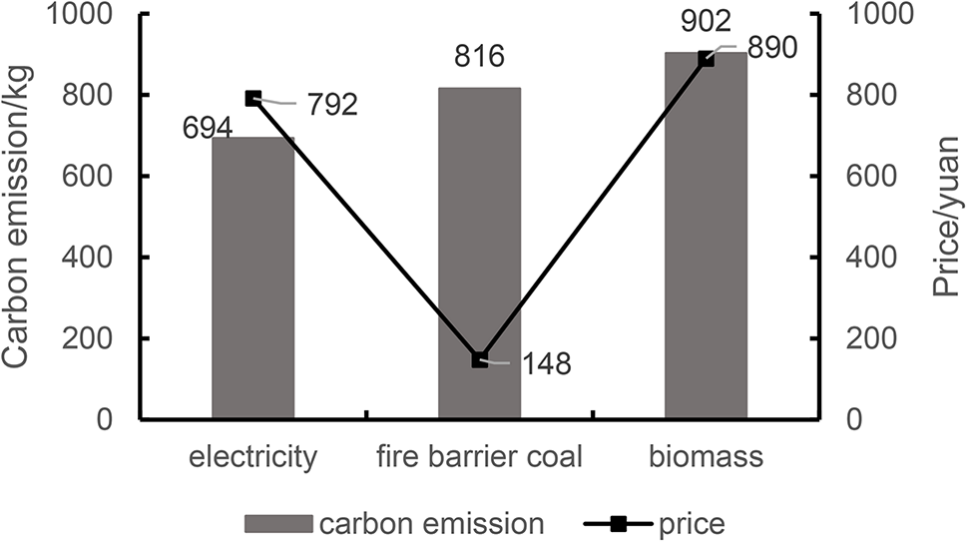
Carbon emissions and economic costs of different heating methods.

Simulation data and results analysis: From the above simulations, during the operation of the building, the carbon emission is the smallest when the heating mode is electricity, and the carbon emission is the largest when the biomass mode is wood. The use of household coal is the least economical. The carbon emissions simulation data of different heating methods are shown in Fig. 19.

#### Economic feasibility analysis

- Electric heating method. The initial investment cost should consider the purchase and installation costs of electric heating equipment; The known operating cost is 791.57 yuan per year (based on the electricity price of 0.6 yuan/kW·h), and the impact of electricity price fluctuations on long-term operating costs should be considered. The impact of equipment life on long-term maintenance costs should be considered, and the cost of regular maintenance, fault repair, and replacement of parts should be considered; The carbon emission is small, which is conducive to environmental protection and sustainable development.
- Household coal heating method. The initial investment cost should consider the purchase and installation of coal-fired stoves and the construction cost of coal storage facilities; Consider the impact of the efficiency and durability of coal-fired stoves on the initial investment; The coal cost is known to be 147.9 yuan per year (based on the coal price of 510 yuan/t), and the impact of coal price fluctuations on operating costs is analysed. Consider the cost of coal storage, transportation, and disposal of cinder; the cost of regular maintenance, breakdown repairs, and replacement of spare parts for coal-fired stoves; Consider the environmental pollution and remediation costs caused by coal burning; Carbon emissions are relatively high, which is not conducive to environmental protection; Consider the potential negative social and economic impacts of environmental pollution.
- Biomass (wood, hay, etc.) heating methods. The purchase and installation of biomass combustion equipment and the construction of biomass storage facilities; Consider the impact of the efficiency and durability of biomass burning equipment on the initial investment; The known timber cost is 889.8 yuan per year (based on the timber price of 839.44 yuan/t); Analyse the impact of timber price fluctuations on operating costs; Consider the cost of biomass collection, transportation, and disposal of waste; Biomass, as a renewable energy source, has long-term sustainability; consider the impact of the availability and stability of biomass resources on economic viability; Carbon emissions are high, but there are still certain environmental advantages compared with coal; Consider the specific effects of biomass burning on the environment, such as air quality and ecosystems.

In summary, different heating methods have their advantages and disadvantages in terms of economic viability. In addition to cost comparisons, factors such as initial investment, operating costs, maintenance costs, policy subsidies, tax incentives, environmental benefits, and sustainability should be fully considered during the optimization process.

#### Return on investment (ROI) and long-term impact

- Electric heating. Assuming that the initial investment cost is 5000 yuan, the operating cost of the electric heating mode before optimization is 791.57 yuan/year (based on the electricity price of 0.6 yuan/kW·h). Due to the energy saving of 56.5% after taking optimization measures, the optimized operating cost is 791.57 × (1-56.5%) = 791.57 × 0.435 ≈ 344.42 yuan/year, and the annual saving is 791.57-344.42 = 447.15 yuan/year. The payback period refers to the time from the beginning of the investment to the recovery of the initial investment cost through the saved operating cost, T = 5000/447.15 ≈ 11.18 years. ROI is usually expressed as an annual percentage, ${\text{ROI}} = \frac{{{\text{Annual}}\,{\text{savings}}}}{{{\text{Initial}}\,{\text{investment}}\,{\text{cost}}}} \times 100\% = \frac{{447.15}}{{5000}} \times 100\% = 8.94\%$. Under the assumption that the initial investment cost is 5000 yuan, the return on investment of 56.5% energy saving after optimization is about 8.94%, and the payback period is about 11 years. This means that after about 11 years, the initial investment cost can be recovered by saving operating costs.
- Household coal heating. Assuming that the initial investment cost is 2000 yuan, the operating cost of the household coal-fired heating mode before optimization is 147.9 yuan per year (based on the coal price of 510 yuan/t). Due to the energy saving of 56.5% after taking optimization measures, the optimized operating cost is 147.9 × (1-56.5%) = 147.9 × 0.435 ≈ 64.34 yuan/year, and the annual saving is 147.9-64.53 = 83.37 yuan/year. The payback period refers to the time from the beginning of the investment to the recovery of the initial investment cost through the saved operating cost, T = 2000 / 83.37 ≈ 23.99 years. ROI is usually expressed as an annual percentage, ${\text{ROI}} = \frac{{83.37}}{{2000}} \times 100\% = 4.17\%$. Under the assumption that the initial investment cost is 2000 yuan, the return on investment of 56.5% energy saving after optimization of household coal-fired heating mode is about 4.17%, and the payback period is about 24 years. This means that after about 24 years, the initial investment cost can be recovered by saving operating costs.
- Biomass heating. Assuming that the initial investment cost is 3000 yuan, the operating cost of the biomass heating method before optimization is 889.8 yuan (based on the wood price of 839.44 yuan/t). Due to the energy saving of 56.5% after taking optimization measures, the optimized operating cost is 889.8 × (1-56.5%) = 889.8 × 0.435 ≈ 387.06 yuan/year, and the annual saving is 889.8-387.06 = 502.74 yuan/year. The payback period refers to the time from the beginning of the investment to the recovery of the initial investment cost through the saved operating cost, T = 3000 / 502.74 ≈ 5.97 years. ROI is usually expressed as an annualized percentage, ${\text{ROI = }}\frac{{502.74}}{{3000}} \times 100\% = 16.77\%$. Assuming that the initial investment cost is 3000 yuan, the return on investment of 56.5% energy saving after optimization of biomass heating mode is about 16.77%, and the payback period is about 6 years. This means that after about 6 years, the initial investment cost can be recovered by saving the operating cost.

In summary, the long-term impact of heating methods involves multiple aspects such as economic, environmental, social and sustainability. When deciding these factors should be considered to choose the heating method that best suits local conditions and needs.

## Discussion and outlook

### Discussion

Comparison with similar studies at home and abroad. This study provides some unique insights into the design and optimization of passive low-energy buildings in ethnic minority areas of the Sichuan Plateau. For example, some studies^24^ on building energy consumption in cold or severe cold regions have also highlighted the importance of thermal design of envelopes, such as enhancing the insulation of facades and roofs, and making the most of solar radiation through reasonable window-to-wall ratio design. However, while these studies tend to focus on the universal cold climate zones, this study focuses on the Sichuan Plateau, a region with unique geographical and climatic conditions. Compared with these studies, this study not only analyzes the impact of energy-saving renovation of the envelope on energy consumption, but also deeply explores the role of factors such as building orientation and layout renovation in improving indoor comfort and reducing energy consumption. In addition, this study combined field tests and questionnaires to obtain direct feedback from residents on thermal comfort, which is not common in similar studies.

Comparison with other studies in rural region China. This study focuses on the ethnic minority areas of the Sichuan Plateau, which have a unique geographical, climatic, and cultural background that is significantly different from other rural areas in China. In contrast, studies^36–39^ in most areas of rural China tend to focus more on plains or hilly areas and less on plateaus. This study combines a combination of field tests, questionnaires, and theoretical analysis, as well as software simulations, to comprehensively assess building energy consumption and indoor comfort. This approach is more comprehensive and in-depth than a single research method (i.e., relying solely on simulations and questionnaires). According to the characteristics of the Sichuan Plateau, this study proposes specific design strategies for passive low-energy buildings, such as enhancing the thermal insulation performance of the envelope and setting the window-to-wall ratio reasonably. These strategies are highly targeted and practical and can be directly applied to the renovation and optimization of local dwellings. This study not only focuses on the reduction of building energy consumption, but also deeply analyzes the carbon emissions, economic costs, and feasibility of different heating methods, and provides more comprehensive options for residents. This is likely to be less covered in studies in other rural areas of China.

Comparison with other studies under similar climatic conditions around the world. The climatic conditions in the Sichuan Plateau region are like those in some high-altitude or cold regions around the globe. The passive low-energy building design strategies proposed in this study, such as using solar radiation to increase indoor temperature and improving air circulation through reasonable building orientation and floor plan, may also be applicable under similar climatic conditions. Despite the similar climatic conditions, the Sichuan Plateau region has a unique cultural background and architectural traditions. These characteristics are fully considered in the design and optimization process of this study, which makes the research results more regional and cultural. In contrast, other global research may focus more on universal design principles and technical approaches^5–9^; This study not only focuses on the specific situation of the Sichuan Plateau but also explores the generalizability of passive low-energy building design from a broader perspective, which provides a useful reference for the renovation and optimization of residential buildings in other regions.

### Limitation and outlook

1. For the study of the dynamic thermal process of the envelope materials, the influence of two particularly important nonlinear heat transfer processes, namely the phase change heat storage material (PCM) and the moisture content of the building envelope structure, on the heat transfer has not been considered. These factors play a key role in building energy efficiency, indoor thermal comfort, and long-term durability. Therefore, research not only has theoretical value but also has a profound impact on practical applications.
2. This paper only analyzes the thermal design and climate of traditional houses in Wenchuan County, which does not apply to the new residential buildings in Wenchuan County, which are facing new challenges in terms of thermal performance, energy utilization, indoor thermal environment, and personnel comfort, etc., and further research is needed on this type of buildings, which not only helps to improve the energy efficiency and comfort of buildings but also helps to promote the sustainable development of local passive buildings.
3. Given the unique climatic conditions of the western Sichuan Plateau, it is recommended to establish a more realistic indoor thermal comfort standard based on the long-term living habits and physiological adaptability of residents.
4. Due to time constraints, this paper only analyzes and simulates the indoor thermal environment of a traditional house in a village in summer and the building energy consumption in winter. To comprehensively evaluate and optimize the building thermal environment of the traditional houses in the western Sichuan Plateau, relevant field measurements and analyses should be carried out in winter and other seasons. Establish a long-term monitoring and evaluation mechanism for the indoor thermal environment, building energy consumption, and energy-saving effect of traditional houses in the western Sichuan Plateau, to provide data support for follow-up research and policy formulation.

Although this study has achieved certain results, there are still some limitations that require critical comparison with similar studies. For example, some studies have used more sophisticated building energy simulation software to more accurately predict the impact of different design parameters on energy consumption. In addition, some studies have considered a wider variety of renewable energy uses, such as solar photovoltaics and thermal energy systems, while this study has focused on passive design strategies.

In the follow-up study, the comprehensive application of passive low-energy buildings and renewable energy utilization in the Sichuan Plateau can be further explored, and the impact of different design strategies on energy consumption, indoor comfort, and economy can be more comprehensively evaluated. In addition, policymakers should introduce special support policies to encourage the adoption of passive low-energy building design in ethnic minority areas of the Sichuan Plateau, select representative areas for demonstration project construction, and demonstrate the practical application effect of passive low-energy buildings in the Sichuan Plateau. Combined with the climatic characteristics and architectural traditions of the Sichuan Plateau, the formulation and improvement of technical standards and specifications for passive low-energy buildings applicable to the region will help ensure the scientific and rational design of buildings and improve the energy efficiency and comfort of buildings. Supporting innovative practices that combine traditional practices with modern technology, while maintaining the look and feel of traditional dwellings, introduce modern energy-efficient materials and construction techniques to improve the energy efficiency and durability of buildings. In the future, researchers should explore how to combine traditional practices in ethnic minority areas of the Sichuan Plateau with modern technology to create architectural designs that are both energy-efficient and culturally appropriate. In the course of the research, long-term monitoring and data analysis are used to focus on the long-term performance and sustainability of buildings and jointly promote the sustainable development of energy conservation and emission reduction goals of the region.

## Conclusions

This paper conducted a survey and field test on the traditional dwellings in the Sichuan Plateau and conducted a questionnaire survey on the residents in the form of thermal sensation voting. Combined with theoretical analysis and software simulation, building orientation, plane layout and envelope structure thermal design before and after optimization are analyzed. Additionally, the carbon emissions and economic suitability of different heating methods were evaluated. The following conclusions are obtained:

1. The site measurements proved that the indoor humidity of the existing traditional houses in the western Sichuan Plateau is high, and the thermal environment doesnot meet the requirement of local standard.
2. Combined with the research and simulation results, the study found that there is a great potential for energy saving and improving indoor comfort, such as enhancing the thermal insulation performance of the building envelope, increasing south-facing window-to-wall ratio. In the winter heating building model data, compared with the heat load before optimization, the energy saving reaches about 56.5%.
3. Building plane should be carefully designed. The formation of draught through the hall improves the air circulation, reduces the indoor relative humidity, and keeps the indoors cool and comfortable in summer. Additionally, increasing a waterproof and moisture-proof layer can significantly reduce indoor relative humidity.
4. It shows that building orientation is an important factor affecting energy consumption, and a linear relationship is found between building energy consumption and orientation: y = 9.0129x + 2076.4, R^2^ = 0.8786. Other factors should be considered in combination with building orientation to achieve the best energy-saving effect.
5. During the operation of the building, with calculated annual heating energy consumption, the carbon emission is the smallest with the electric heating mode, and the largest with the wood and other biomass methods; The use of domestic coal is the least economical. When choosing a heating method, it is necessary to consider factors such as carbon emissions, economic costs, local resource conditions, and environmental policies.

## Data Availability

The data used to support the findings of this study are included within the article.
